# Efficacy Confirmation Test of Black Cumin (*Nigella sativa* L.) Seeds Extract Using a High-Fat Diet Mouse Model

**DOI:** 10.3390/metabo13040501

**Published:** 2023-03-30

**Authors:** Khawaja Muhammad Imran Bashir, Joo Wan Kim, Jong-Kyu Kim, Yoon-Seok Chun, Jae-Suk Choi, Sae-Kwang Ku

**Affiliations:** 1 German Engineering Research Center for Life Science Technologies in Medicine and Environment, Busan 46742, Republic of Korea; imran.bashir@lstme.org; 2 Department of Companion Animal Health, Daegu Haany University, Gyeongsan 38610, Republic of Korea; dvm_jkkim@dhu.ac.kr; 3 AriBnC, Ltd., Yongin 16985, Republic of Korea; jkkim@aribnc.com (J.-K.K.); ceochun@aribnc.com (Y.-S.C.); 4 Department of Seafood Science and Technology, The Institute of Marine Industry, Gyeongsang National University, Tongyeong 53064, Republic of Korea; 5 Department of Anatomy and Histology, College of Korean Medicine, Daegu Haany University, Gyeongsan 38610, Republic of Korea

**Keywords:** black cumin, metabolic disorder, NAFLD, obese diabetic mice, type 2 diabetes

## Abstract

To deal with the adverse effects associated with the use of currently available treatments for metabolic disorders, such as type 2 diabetes, there is a need to find an alternative drug compound. In the present study, we investigated the therapeutic potential of black cumin (*Nigella sativa* L.) seeds extract (BCS extract) for type 2 diabetes using a 45% Kcal-fed obese mouse model. The BCS extract at different doses (400–100 mg/kg) showed a dose-dependent improvement tendency in high-fat diet (HFD)-induced obesity, non-alcoholic fatty liver disease (NAFLD), hyperlipidemia, and diabetic nephropathy compared to the metformin (250 mg/kg). In particular, BCS extract at a dose of 200 mg/kg significantly inhibited the HFD-induced metabolic conditions. The oral administration of BCS extract (200 mg/kg) significantly inhibited the oxidative stress through lipid peroxidation, normalized the activity of sugar metabolism-related enzymes and the expression of genes involved in fat metabolism, and inhibited insulin resistance through glucose and fat metabolism by regulating the 5’-AMP-activated protein kinase (AMPK) expression. Furthermore, BCS extract (200 mg/kg) showed renal damage improvement effects compared to the metformin (250 mg/kg). The results clearly show that BCS aqueous extract at an appropriate concentration could help in the treatment of metabolic disorders, and BCS aqueous extract can be used as a functional food for various diabetic complications, such as obesity, diabetes, and NAFLD.

## 1. Introduction

Obesity causes various lethal metabolic disorders, such as type 2 diabetes, hypertension, and cardiovascular diseases [1]. In obesity, adipocytes secrete adipokines, which cause chronic inflammation and associated metabolic disorders [2]. When insulin resistance is induced in adipocytes, excess fatty acid binding and transport proteins lead to an increase in fatty acid uptake by non-adipocytes [2]. The accumulation of free fatty acids in the liver causes insulin resistance, and a large amount of glucose begins to liberate [3]. The accumulation of triglyceride (TG) in hepatocytes causes non-alcoholic fatty liver disease (NAFLD), which results in fat accumulation in hepatocytes and finally causes fibrosis due to the necrosis of hepatocytes [4]. Thus, a balance in hepatocytes fat decomposition and synthesis provides an important therapeutic target that can prevent the induction of NAFLD and insulin resistance caused by a metabolic syndrome [5].

A high-fat diet (HFD) can induce severe obesity in rodent mice. In earlier studies, significant insulin resistance, hyperglycemia, hyperlipidemia, mild diabetic nephropathy, and nonalcoholic steatohepatitis (NASH) have been observed in HFD-fed obese mice compared to normal pellet diet (NFD)-fed mice [6,7,8,9]. The extensively used HFD-fed animal model for studying the efficacy of functional foods causes obesity and hyperglycemia at an appropriate level and prevents metabolic syndromes including NAFLD [8,9,10]. Therefore, in this study, a 45% Kcal HFD-fed mouse model was used to evaluate the dose-dependent pharmacological effects of a new candidate for metabolic syndromes, particularly obesity.

Currently, various treatments for metabolic syndromes have been developed; however, their use is restricted due to the associated adverse effects [11]. Therefore, the development of alternative medicines from natural sources with few side effects and a higher effectivity is being actively tried [8,9,10]. Recently, the control of oxidative stress, which is a major etiology of diabetes and related complications, along with blood sugar control, has emerged as the most essential method for diabetes treatment [8,9,10,11]. Therefore, the development of more effective α-glucosidase blockers or antioxidants with few side effects is being actively tried [8,9,12,13]. Among those, metformin is a representative oral biguanide-based antidiabetic drug and a well-known 5’-AMP-activated protein kinase (AMPK) activator [14,15]. It lowers the onset of cardiovascular disease, one of the serious side effects caused by diabetes, and the secretion of enzyme granules from the pancreas, which are directly involved in the digestion and decomposition of fats [8,9,10]. It also regulates the activity of enzymes related to hepatic glucose metabolism [16,17]. In the present study, 250 mg/kg of metformin was used as a standard drug based on our earlier drug efficacy studies [8,9,10].

The black cumin (*Nigella sativa* L.) belongs to the family Ranunculaceae, and its seeds are commonly used as food spices [18,19]. In Islamic culture, they are called “El Habba Saouda (seeds of blessing)” and are known as a medicinal herb traditionally used for all ailments except for death [19]. Black cumin contains a wide variety of active ingredients, such as polyphenols, flavonoids, saponins, and alkaloids [20]. Thymoquinone, present in *N. sativa*, is an especially important pharmacologically active compound and has been proven to have anti-rheumatoid arthritis, anti-inflammatory, and anti-cancer properties [21]. Furthermore, it has shown anti-diabetic and anti-obesity effects by increasing phosphorylated Sirtuin 1 (SIRT1) in the liver and muscle and AMPKα in the muscle [22].

The antioxidant [23], anti-inflammatory [24], immunomodulatory [25], anticancer [26], antibacterial [27], antifungal [28], and hypersensitivity inhibition properties [29] of black cumin have been experimentally proven. As black cumin is also known for its glycemic control activity through its glycated hemoglobin inhibitory effect [30], its potential as a preventive and therapeutic agent for diabetes is attracting attention [31,32]. In a recent study, Esmail et al. [33] investigated the anti-diabetic potential of *N. sativa*, where they focused only on blood lipid- and liver-related markers and liver biopsy. Although the bio-efficiency of thymoquinone or various organic solvent extracts of black cumin has been extensively reported, the efficacy study of supercritical water extract is not available. Thus, in the present study, we focus on the overall efficacy of black cumin seeds supercritical water extract (BCS extract) for anti-obesity, anti-diabetes, and related complications to validate the previously available effects of *N. sativa*. As part of the development of a natural product-derived functional food material for improving obesity and diabetes-related complications, the dose-dependent effects of BCS aqueous extract on improving obesity and diabetes-related complications were evaluated using an HFD-fed mice model, which is a commonly used experimental animal model for mild type 2 diabetes and obesity [7,8,9].

## 2. Materials and Methods

### 2.1. Test Material

The powdered black cumin (*Nigella sativa* L.) seeds extract was provided by AriBnC, Yongin, Korea. Briefly, crushed black cumin seeds were mixed with 0.12% (*w*/*w*) rosmarinic acid, and the seeds extract was prepared at 300 bars using the super critical fluid extraction technique, as reported by Choi et al. [34]. After collecting the seeds extract, black cumin dietary fiber was additionally obtained from the remaining free-flowing powder. The obtained black cumin seeds supercritical water extract (30–35%; *w*/*w*) was mixed with 50–52% (*w*/*w*) black cumin dietary fiber and 15–16% (*w*/*w*) magnesium carbonate and homogenized to prepare the raw material (black cumin seeds extract; BCS extract) used in this study. The extract was further lyophilized at −85 °C into a light brown powder which showed solubility up to 40 mg/mL when dissolved in distilled water. The BCS extract was stored at −20 °C for later use.

### 2.2. High-Performance Liquid Chromatography Analyses

The amount of thymoquinone in BCS extract was quantified using Agilent HPLC 1100 series (Agilent Technologies, Inc., Santa Clara, CA, USA) equipped with a UV-visible absorbance detector (Agilent Technologies, Inc.) and Capcell Pak C18 MG II column (4.6 mm × 250 mm, 5 µm; Osaka Soda Co. Ltd., Osaka, Japan). BCS extract and the standard thymoquinone (Wuhan ChemNorm Biotech Co., Ltd, Wuhan, China) were dissolved in acetonitrile and filtered using 0.45 µm membrane filter before injection. The column was kept at 30 °C during analysis, and thymoquinone was analyzed at 254 nm. A mobile phase consisted of a mixture of distilled water and acetonitrile (18:82). Ten microliters of each sample were injected at a flow rate of 0.9 mL/min and the results were quantitatively analyzed.

### 2.3. Experimental Animals

A total of 80 six-week-old female SPF/VAF CrlOri:CD1 (ICR) mice were obtained from OrientBio (Seungnam, Korea) and acclimatized to the laboratory conditions for seven days. Changes in lifestyle and a lack of exercise during adolescence have been linked with higher risks of obesity in young girls [35]. In females, obesity causes menstrual irregularities, hormonal imbalance, and social problems including social maladjustment and excessive medical expenses [36,37]; thus, a female mouse model was used in this study. The experimental animals (*n* = 68) were fed on HFD (Table S1; Research Diet, New Brunswick, NJ, USA) for one week, and the NFD control (*n* = 12) mice were fed with a commercial normal pellet diet (Purinafeed, Seungnam, Korea). A total of six groups (50 HFD-fed mice with an average weight of 31.68 ± 1.38 g and 10 NFD-fed mice with an average weight of 28.99 ± 1.58 g) were used in this study. All experimental animals were treated following the animal experiment and ethics standards under the approval of the Laboratory Animal Ethics Committee of Daegu Haany University (Approval No.: DHU2020-055; Approved on 7 September 2020).

The following experimental animal groups (*n* = 10 mice in each group) were selected for this study.

1. NFD control = 10 mL/kg of vehicle (distilled water)-administered mice with NFD supply
2. HFD control = 10 mL/kg of vehicle (distilled water)-administered mice with HFD supply
3. MET_250_ = Control drug—metformin (250 mg/kg)-administered mice with HFD supply
4. BCS_400_ = BCS extract (400 mg/kg)-administered mice with HFD supply
5. BCS_200_ = BCS extract (200 mg/kg)-administered mice with HFD supply
6. BCS_100_ = BCS extract (100 mg/kg)-administered mice with HFD supply

### 2.4. Dose Selection of the Test Substance

In this study, the oral dose levels of BCS extract were selected as 400, 200, and 100 mg/kg based on the United States Food and Drug Administration (US-FDA) guidelines for estimating the safe starting dose in initial clinical trials [38] and calculated by the following formula [39] in Equation (1).

(1) $$Human\;equivalent\;dose\left(\frac{2\;g}{60\;\mathrm{kg}}\right)=Animal\;dose\left(\frac{\mathrm{mg}}{\mathrm{kg}}\right)*\frac{3\left(animal-\mathrm{km}\right)}{37\left(human-\mathrm{km}\right)}$$

About 2 g/60 kg = 411 (mg/kg) × 3/37. The calculated high dose of 400 mg/kg was divided by 2, and the medium (200 mg/kg) and low (100 mg/kg) doses of BCS extract were decided for this study. BCS extract suspended in sterile distilled water was orally administered at a volume of 10 mL/kg [40]. In addition, 250 mg/kg of metformin was used as a standard drug [8,9,10].

### 2.5. Test Material Administration

The experimental animals were supplied with HFD from one week before starting the test substance administration and during the whole period of test substance administration, whereas the NFD control groups were supplied with the NFD. After a one-week HFD adaptation period, BCS extract was administered orally at a dose of 400, 200, and 100 mg/kg body weight once daily for 84 days using a 1 mL syringe connected with a metal sonde. In addition, metformin was orally administered at a dose of 250 mg/kg once daily for 84 days. In the NFD and HFD control groups, only sterilized distilled water was orally administered instead of the test material.

### 2.6. Observation Items

Blood sugar lowering (anti-diabetic), hepatoprotection, renal protection, anti-hyperlipidemia, anti-obesity, antioxidant effects, hepatic lipid peroxidation, liver enzyme activity related to glucose metabolism, and effects on fat metabolism-related genes expression were studied using macroscopic, hematological, histobiochemical, histopathological, biochemical, and real-time RT-PCR tests, respectively, as reported in our previous studies [7,8,9,10]. Each method is briefly described below.

#### 2.6.1. Blood Sugar Lowering (Anti-Diabetic) Effects

Whole blood was collected from the caudal vena cava, and after separating plasma, the blood sugar was measured using an automated blood sugar analyzer (Dri-chem, Fuji Medical System Co. LTD, Tokyo, Japan). The whole blood and serum were separated by centrifugation at 16,000× *g* for 10 min, and the amount of glycated hemoglobin A1c (HbA1c) was measured using automated HbA1c measuring equipment (Model Easya1c; Infopia, Anyang, Korea). Blood insulin levels were measured using a mouse insulin ELISA kit (Alpco Diagnostics, Windham, NH, USA). Organs were separated under isoflurane anesthesia, and pancreatic weight was measured using an electronic balance (Precisa Instruments, Dietikon, Switzerland). The separated pancreatic tissue was fixed on a slide using 10% neutral formalin solution and stained with hematoxylin and eosin (H&E; Sigma-Aldrich, St. Louis, MO, USA). The number and diameter of pancreatic glands were noted. The glucagon and insulin immune responses were analyzed using anti-glucagon (Abcam, Cambridge, UK) and anti-insulin (Abcam, Cambridge, UK) antibodies, respectively. The tissues were immunoassayed by the avidin-biotin-peroxidase (ABC) method [41] and observed under a microscope (Eclipse 80i; Nikon Corp., Tokyo, Japan).

#### 2.6.2. Hepatoprotective Effects

Organs were separated under isoflurane anesthesia, and liver weights were measured using an electronic balance (Precisa Instruments, Dietikon, Switzerland). An automated blood analyzer (Dri-chem, Prague, Czech Republic) was used to analyze serum alanine transaminase (ALT), aminotransferase (AST), alkaline phosphatase (ALP), gamma-glutamyl transferase (GGT), and lactate dehydrogenase (LDH) content. After fixing the liver tissue in 10% formalin and H&E staining, the degree of damage to the liver tissue and the size of the liver cells were estimated by an automated image analysis—iSolution FL ver. 9.1 (IMT i-solution Inc., Vancouver, QC, Canada), as reported in our previous studies [10,41].

#### 2.6.3. Nephroprotective Effects

Kidney weights were recorded by an electronic balance (Precisa Instruments, Dietikon, Switzerland). Kidney tissues were fixed in 10% formalin, and histopathological changes in kidney tissue were observed under a microscope (Nikon Corp., Tokyo, Japan) stained after H&E staining. Creatinine and blood urea nitrogen (BUN) contents were analyzed by an automated blood analyzer (Dri-chem, Prague, Czech Republic).

#### 2.6.4. Antihyperlipidemic Effects

Changes in total cholesterol (TC), TG, high-density lipoprotein (HDL), and low-density lipoprotein (LDL) content were analyzed using an automated blood analyzer (Dri-Chem). The fecal lipid contents were measured on the poop. The poop in the cage was collected and analyzed at 8 a.m. the next day after the last test substance administration. The TG and TC contents in feces were obtained by extracting fat by the chloroform:methanol method of Folch et al. [42]. The extracted fats were analyzed using a total cholesterol assay kit (Cell Biolabs, San Diego, CA, USA) and a total glyceride colorimetric assay kit (Cayman, Ann Arbor, MI, USA).

#### 2.6.5. Anti-Obesity Effects

Dual energy X-ray absorptiometry (DEXA) was used to analyze the amount of abdominal and body fat accumulation, according to the method of Chen et al. [43]. Fat organs were removed under anesthesia, and periovarian and abdominal wall fat accumulation was measured using an electronic balance (Precisa Instruments, Dietikon, Switzerland). The excised pancreatic tissue was fixed in 10% formalin, and after staining with H&E, histopathological changes in the exocrine pancreas, changes in the thickness of the periovarian and abdominal wall accumulated fat, average white adipocyte size, and the proportion of zymogen granules in pancreatic exocrine secretion were analyzed using a microscope (Nikon Corp., Tokyo, Japan).

#### 2.6.6. Antioxidant Effects

Lipid peroxidation (Malondialdehyde; MDA content) analysis of the homogenized liver tissue was carried out by a thiobarbituric acid test [44], and the readings were recorded at 525 nm by a UV/Vis spectrophotometer (OPTIZEN POP, Mecasys, Daejeon, Korea). Changes in the endogenous antioxidant glutathione (GSH) content were measured at 412 nm after reaction with 2-nitrobenzoic acid [45]. Changes in antioxidant defense enzyme–catalase (CAT) activity, the degree of decomposition of 1 nM of H_2_O_2_ (25 °C, pH 7.8) in 1 min, were measured by the method of Bolzán et al. [46]. The superoxide dismutase (SOD) activity was analyzed using the formazan dye method [47], and the readings were recorded at 560 nm using a UV/Vis spectrophotometer (OPTIZEN POP).

#### 2.6.7. Changes in Glucose Metabolism-Related Enzymes Activity

The activity of the glucokinase (GK) enzyme was measured by the Davidson and Arion [48] method. Briefly, the reaction solution (100 mM KCl, 50 mM sodium HEPES (pH 7.4), 50 mM NAD+, 10 mM glucose, 7.5 mM MgCl_2_, 2.5 mM dithioerythritol, 10 mg/mL albumin, and 4 units of glucose-6-phosphate dehydrogenase) was mixed with 10 μL hepatic tissue homogenate and reacted at 37 °C for 10 min. After adding 10 μL of 5 mM ATP in the reaction mixture, the reaction mixture was incubated again at 37 °C for 10 min, and the readings were recorded at 340 nm using a UV/Vis spectrophotometer (OPTIZEN POP). Glucose-6-phosphatase (G6pase) enzyme activity was measured by the method of Alegre et al. [49]. Briefly, the reaction solution (765 μL of 131.58 mM sodium HEPES (pH 6.5), 100 μL of 265 mM glucose-6-phosphate, 100 μL of 18 mM EDTA (pH 6.5), 10 μL of 0.2 M NADP+, 0.6 IU/mL mutarotase, and 0.6 IU/mL glucose dehydrogenase) was mixed with 10 μL hepatic tissue homogenate and incubated at 37 °C for 4 min. The readings were measured at 340 nm using a UV/Vis spectrophotometer (OPTIZEN POP). Phosphoenolpyruvate carboxykinase (PEPCK) activity was measured by the method of Punekar and Lardy [50]. Briefly, the reaction solution (500 mM NaHCO_3_, 200 mM PEP, 100 mM IDP, 72.92 mM sodium HEPES (pH 7.0), 25 mM NADH, 10 mM MnCl_2_, 10 mM dithiothreitol, and 7.2 units of malic dehydrogenase) was mixed with 10 μL hepatic tissue homogenate, and the readings were recorded at 340 nm using a UV/Vis spectrophotometer (OPTIZEN POP).

#### 2.6.8. Gene Expression Related to Fat Metabolism

The mRNA expressions of liver acetyl-CoA carboxylase 1 (ACC1), AMP-activated protein kinase (AMPKα1 and AMPKα2), adipose tissue leptin, uncoupling protein 2 (UCP2), adiponectin, markers for liver development (C/EBPα and C/EBPβ), sterol regulator element binding protein (SREBP1c), cell surface death receptor (FAS), and peroxisome proliferator-activated receptors (PPARα and PPARγ) were obtained using a real-time RT-PCR (Bio-Rad, Hercules, CA, USA), as reported in our previous studies [7,8,9]. The expression patterns were compared with the mRNA expressions of glyceraldehyde-3-phosphate dehydrogenase (GAPDH), used as a standard, and gene expressions were calculated by the comparative CT method of Schmittgen and Livak [51]. The oligonucleotides used in this study are listed in Table S2.

### 2.7. Statistical Analyses

The numerical values are expressed as the means ± standard deviation (S.D.) of ten mice. Statistical comparisons between different dose groups were performed using the Levene test, Dunnett’s T3 (DT3) test, and Tukey’s Honest Significant Difference (THSD) test, as reported in our earlier studies [7,8,9,10]. Briefly, after one-way analysis of variance (ANOVA), if no variation was observed, the THSD test was performed, and if the samples showed variances, then the DT3 test was performed. Statistical significance was analyzed using SPSS ver. 18 (IBM-SPSS Inc., Armonk, NY, USA), and statistical differences were considered significant at *p* < 0.05.

## 3. Results

### 3.1. Concentration of Thymoquinone in BCS Extract 

In HPLC analysis, retention time of the standard thymoquinone was 4.207 min and the BCS extract showed a similar retention time of 4.214 min, thus both samples were considered as same components. As a result, the thymoquinone concentration in the BCS extract was measured according to the standard compound’s concentration comparison formula and thymoquinone concentration of 58.53 mg/g (5.853%) was found in the BCS extract as confirmed by HPLC analyses (Figure S1).

### 3.2. Anti-Obesity Effects

#### 3.2.1. Change in Body Weight

Experimental animals with a constant weight gain were selected after acclimatization, showing an average body weight of 31.68 ± 1.38 g in the HFD-fed group and 28.99 ± 1.58 g in the NFD-fed group. The HFD control group showed a significant (*p* < 0.01) increase in body weight after six days of HFD feeding and throughout the experimental period of 84 days, whereas the BCS_400_, BCS_200_, BCS_100_, and MET_250_ groups showed significant (*p* < 0.01; *p* < 0.05) weight losses after 21, 28, 21, and 28 days, respectively, as compared to the HFD control group. Particularly, BCS extract (200 mg/kg) significantly inhibited an increase in HFD-induced body weight and weight gain, comparable to the MET_250_ (Table S3).

#### 3.2.2. Changes in Average Feed Intake

The HFD control group showed a significant (*p* < 0.01) decrease in average feed intake as compared to the NFD control group. However, no notable changes in average feed intake were observed in all experimental groups, including the BCS_400_, as compared to the HFD control group (Table S3).

#### 3.2.3. Changes in Body and Abdominal Fat Mass

The HFD control mice showed significant (*p* < 0.01) increases in body and abdominal fat mass accumulations as compared to the NFD control mice, whereas the oral administration of BCS extract (400–100 mg/kg) significantly (*p* < 0.01; *p* < 0.05) and dose-dependently reduced the abdominal and body fat mass accumulations. Particularly, the BCS_200_ group significantly inhibited the increase in HFD-induced fat mass accumulation, comparable to the MET_250_ (Figure S2; Table 1).

#### 3.2.4. Changes in Fat Weight

The HFD control group showed significant (*p* < 0.01) increases in the absolute and relative weights of abdominal and periovarian wall fat accumulations as compared to the NFD control group, whereas the oral administration of BCS extract (400–100 mg/kg) significantly and dose-dependently reduced the abdominal and periovarian wall fat weights. Particularly, BCS extract (200 mg/kg) significantly inhibited the absolute and relative weight gains of HFD-induced abdominal and periovarian wall fat accumulations, comparable to the MET_250_ (Figure S2; Table 1).

#### 3.2.5. Histopathological Changes in Periovarian and Abdominal Wall Fat Accumulation

The HFD control group showed significant adipocyte hypertrophy and a significant (*p* < 0.01) increase in the diameter and thickness of the abdominal and periovarian wall fat accumulated adipose tissue as compared to the NFD control mice. However, all three doses of BCS extract (400, 200, and 100 mg/kg) significantly (*p* < 0.01) reduced the diameter and thickness of abdominal and periovarian wall fat accumulation. Particularly, BCS extract (200 mg/kg) inhibited the increase in HFD-induced abdominal and periovarian wall fat accumulation as compared to the MET_250_ (Figure S3; Table 2).

#### 3.2.6. Histopathological Changes in the Pancreatic Exocrine Zymogen Granules

The HFD supply significantly (*p* < 0.01) decreased the proportion of zymogen granules in pancreatic exocrine as compared to the NFD-supplied mice. However, all three doses of BCS extract (400, 200, and 100 mg/kg) significantly increased the ratio of zymogen granules. Particularly, BCS extract (200 mg/kg) showed an inhibitory effect on the reduction in the HFD-induced ratio of zymogen granules comparable to the MET_250_ (Figure S4; Table 3).

### 3.3. Anti-Diabetic Effects

#### 3.3.1. Changes in Blood Sugar

The HFD supply significantly (*p* < 0.01) increased blood glucose in mice as compared to the NFD control group. However, the BCS extract-administered groups at all three doses (400, 200, and 100 mg/kg) significantly (*p* < 0.01) and dose-dependently decreased the blood glucose. Particularly, BCS extract (200 mg/kg) significantly inhibited the increase in HFD-induced blood glucose as compared to the MET_250_ (Table 4).

#### 3.3.2. Insulin Content and HbA1c Ratio

**Figure 1:**
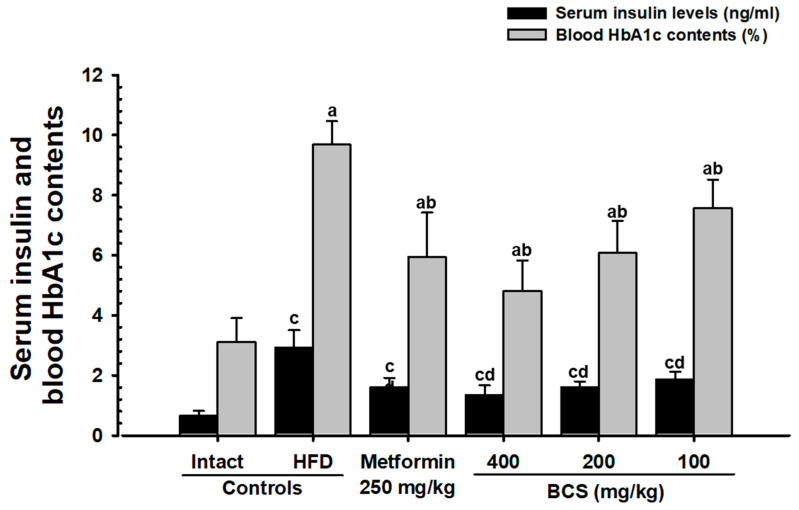
Insulin content and HbA1c ratio in NFD- or HFD-supplied groups. DT3: Dunnett’s T3 test; THSD: Tukey’s Honest Significant Difference test; ^a^ *p* < 0.01 as compared to the NFD control group by the THSD test; ^b^ *p* < 0.01 as compared to the HFD control group by the THSD test; ^c^ *p* < 0.01 as compared to the NFD control group by the DT3 test; ^d^ *p* < 0.01 as compared to the HFD control group by the DT3 test.

The HFD supply significantly (*p* < 0.01) increased the blood insulin content and HbA1c ratio as compared to the NFD control group. However, all three doses (400, 200, and 100 mg/kg) of BCS extract significantly (*p* < 0.01) and dose-dependently decreased the blood insulin content and HbA1c ratio. Particularly, BCS extract (200 mg/kg) significantly inhibited the HFD-induced increase in the blood insulin and HbA1c ratio as compared to the MET_250_ (Figure 1).

#### 3.3.3. Changes in Pancreatic Weights

The HFD-supplied controls showed significant (*p* < 0.01) decreases in pancreatic relative weights as compared to the NFD control group, whereas, the oral administration of BCS extract at all three doses (400, 200, and 100 mg/kg) significantly increased the relative pancreatic weights. Particularly, BCS extract (200 mg/kg) significantly inhibited the reduction in the relative pancreatic weights as compared to the MET_250_. No notable changes in the absolute pancreatic weight were observed in the HFD-supplied control mice as compared to the NFD-supplied control mice. Furthermore, the absolute pancreatic weights observed in the BCS (250 mg/kg)-administered mice were also not significant as compared to the HFD-supplied control mice (Table 5).

#### 3.3.4. General Histopathological Changes in Pancreatic Islets

The HFD control group showed a significant proliferation of pancreatic islets and a significantly (*p* < 0.01) increased average diameter and number of pancreatic islets as compared to the NFD control group. However, all three doses of BCS extract (400, 200, and 100 mg/kg) significantly (*p* < 0.01) decreased the diameter and number of pancreatic islets. Particularly, an oral dose of BCS extract (200 mg/kg) significantly inhibited the increase in the diameter and number of HFD-induced pancreatic islets as compared to the MET_250_ (Figure S5; Table 2).

#### 3.3.5. Immunohistochemical Changes in Pancreatic Islets

The HFD control supply in control mice significantly (*p* < 0.01) increased the number of glucagon and insulin immune-reactive cells and the insulin/glucagon cell ratio as compared to the NFD control mice. However, all three doses of BCS extract (400, 200, and 100 mg/kg) significantly (*p* < 0.01) decreased the number of glucagon and insulin immune-reactive cells and the insulin/glucagon cell ratio. Particularly, an oral dose of BCS extract (200 mg/kg) significantly inhibited the increase in the number of HFD-induced glucagon and insulin immune-reactive cells and the insulin/glucagon cell ratio, comparable to the MET_250_ (Figure S5; Table 3).

### 3.4. Effects on Hyperlipidemia

#### 3.4.1. TC, TG, and LDL Content in Blood

The HFD-supplied control group showed significantly (*p* < 0.01) increased blood TC, TG, and LDL contents as compared to the NFD control group. However, all three doses of BCS extract (400, 200, and 100 mg/kg) significantly (*p* < 0.01) decreased the TC, TG, and LDL content in blood. Particularly, an oral dose of BCS extract (200 mg/kg) significantly inhibited the HFD-induced increases in the TC, TG, and LDL content in the blood as compared to the MET_250_ (Table 4).

#### 3.4.2. HDL Content in Blood

The HFD-supplied control group showed a significantly (*p* < 0.01) decreased blood HDL content as compared to the NFD control group. However, all three doses of BCS extract (400, 200, and 100 mg/kg) significantly (*p* < 0.01) increased the HDL content in the blood. Particularly, BCS extract (200 mg/kg) showed an inhibitory effect on the reduction in HFD-induced blood HDL content as compared to the MET_250_ (Table 4).

#### 3.4.3. TC and TG Content in Feces

**Figure 2:**
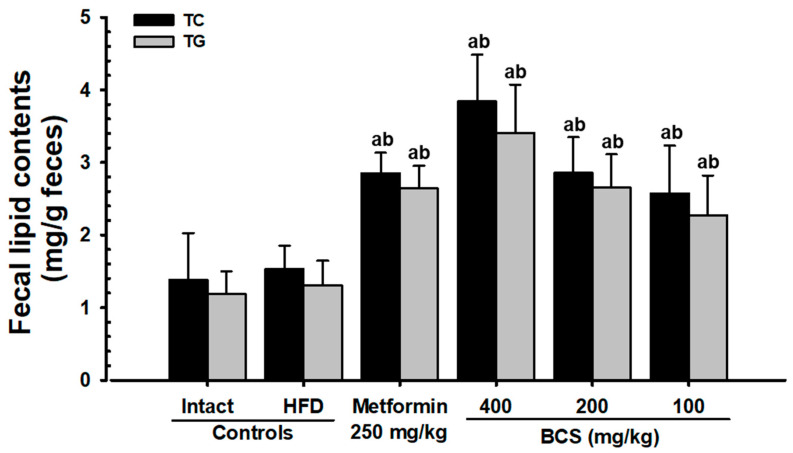
Fecal TC and TG content in NFD- or HFD-supplied groups. DT3: Dunnett’s T3 test; THSD: Tukey’s Honest Significant Difference test; ^a^ *p* < 0.01 as compared to the NFD control group by the THSD test; ^b^ *p* < 0.01 as compared to the HFD control group by the THSD test.

The HFD control group showed a slight increase in the TC and TG content in feces as compared to the NFD control group. However, all three doses of BCS extract (400, 200, and 100 mg/kg) significantly (*p* < 0.01) increased the TC and TG content in feces as compared to the HFD control group. Particularly, BCS extract (200 mg/kg) showed an increasing effect for the TC and TG content in feces as compared to the MET_250_ (Figure 2).

### 3.5. Effects on Liver Damage

#### 3.5.1. Changes in Liver Weight

The HFD supply in control mice significantly (*p* < 0.01) increased the absolute liver weight as compared to the NFD control group. However, all three doses of BCS extract (400, 200, and 100 mg/kg) showed significant (*p* < 0.01) decreases in the absolute liver weights. Particularly, BCS extract (200 mg/kg) inhibited the increase in HFD-induced absolute liver weight as compared to the MET_250_. No significant changes in liver relative weight were observed in the HFD control group as compared to the NFD control group. In addition, changes in the liver relative weights in the BCS (100 mg/kg)-supplied mice were also not significant as compared to the HFD control group (Table 5).

#### 3.5.2. AST, ALT, ALP, LDH, and GGT Content in Blood

The HFD supply in the control group significantly (*p* < 0.01) increased the blood AST, ALT, ALP, LDH, and GGT content as compared to the NFD control group. However, all three doses of BCS extract (400, 200, and 100 mg/kg) showed significant (*p* < 0.01) decreases in the AST, ALT, ALP, LDH, and GGT content in the blood. Particularly, BCS extract (200 mg/kg) inhibited the HFD-induced increases in the AST, ALT, ALP, LDH, and GGT content in the blood as compared to the MET_250_ (Table 6).

#### 3.5.3. Histopathological Changes in the Liver Fat Change Rate and Liver Cell Diameter

The HFD supply in the control group showed fatty liver findings and significantly (*p* < 0.01) increased the liver fat change rate and liver cell diameter as compared to the NFD control group. However, all three doses of BCS extract (400, 200, and 100 mg/kg) significantly (*p* < 0.01) decreased the liver fat change rate and mean hepatocyte diameter. Particularly, BCS extract (200 mg/kg) inhibited the HFD-induced increase in the hepatic fat change rate and mean hepatocyte diameter as compared to the MET_250_ (Figure S6; Table 7).

### 3.6. Effects on Kidney Damage

#### 3.6.1. Changes in Kidney Weight

The HFD supply in the control group significantly (*p* < 0.01) increased the absolute kidney weights as compared to the NFD control group. However, all three doses of BCS extract (400, 200, and 100 mg/kg) showed significant (*p* < 0.01) decreases in the absolute renal weights. Particularly, an oral dose of BCS extract (200 mg/kg) inhibited the HFD-induced increases in absolute renal weight as compared to the MET_250_. No notable changes in relative renal weight were observed in the HFD-control group as compared to the NFD control group. In addition, changes in relative renal weights in all test substance-administered groups including the MET_250_ were also not significant (Table 5).

#### 3.6.2. Blood Creatinine and BUN Content

The HFD supply in the control group significantly (*p* < 0.01) increased the blood creatinine and BUN content compared to the NFD control group. However, all three doses of BCS extract (400, 200, and 100 mg/kg) significantly (*p* < 0.01) increased the blood creeatinine and BUN content. Particularly, an oral dose of BCS extract (200 mg/kg) inhibited the HFD-induced increases in the blood creatinine and BUN content as compared to the MET_250_ (Table 6).

#### 3.6.3. Changes in Renal Histopathology

The HFD supply in the control group showed renal degeneration and significantly (*p* < 0.01) increased the number of degenerative tubules as compared to the NFD control group. However, all three doses of BCS extract (400, 200, and 100 mg/kg) significantly (*p* < 0.01) decreased the number of degenerative tubules. Particularly, BCS extract (200 mg/kg) inhibited the increase in the number of HFD-induced degenerative tubules as compared to the MET_250_ (Figure S7; Table 7).

### 3.7. Effects on the Liver Antioxidant Defense System

#### 3.7.1. Changes in Liver Lipid Peroxidation

The HFD supply in the control group significantly (*p* < 0.01) increased the liver lipid peroxidation as compared to the NFD control group. However, all three doses of BCS extract (400, 200, and 100 mg/kg) significantly (*p* < 0.01) decreased the hepatic lipid peroxidation. Particularly, BCS extract (200 mg/kg) inhibited the HFD-induced increases in liver lipid peroxidation as compared to the MET_250_ (Table 8).

#### 3.7.2. GSH Content in the Liver

The HFD supply in the control group significantly (*p* < 0.01) decreased the GSH content as compared to the NFD control group. However, all three doses of BCS extract (400, 200, and 100 mg/kg) significantly (*p* < 0.01; *p* < 0.05) increased the GSH content in the liver. Particularly, BCS extract (200 mg/kg) inhibited the HFD-induced reduction in the liver GSH content as compared to the MET_250_ (Table 8).

#### 3.7.3. CAT and SOD Activity in Liver Tissue

The HFD supply in the control group significantly (*p* < 0.01) decreased the CAT and SOD activities as compared to the NFD control group. However, all three doses of BCS extract (400, 200, and 100 mg/kg) significantly increased the CAT and SOD activities. Particularly, BCS extract (200 mg/kg) inhibited the HFD-induced reductions in CAT and SOD activities in the liver as compared to the MET_250_ (Table 8).

### 3.8. Activity of Glucose Metabolism-Related Liver Enzymes

#### 3.8.1. GK Activity in the Liver

The HFD supply in the control group significantly (*p* < 0.01) decreased the GK activity in liver tissue as compared to the NFD control group, whereas all three doses of BCS extract (400, 200, and 100 mg/kg) showed significant (*p* < 0.01; *p* < 0.05) increases in the liver GK activity. Particularly, BCS extract (200 mg/kg) inhibited the HFD-induced reductions in GK activity, comparable to the MET_250_ (Table 9).

#### 3.8.2. G6pase and PEPCK Activity in the Liver

The HFD supply in the control group significantly (*p* < 0.01) increased the G6pase and PEPCK activity in the liver tissue as compared to the NFD control group. However, all three doses of BCS extract (400, 200, and 100 mg/kg) significantly (*p* < 0.01) decreased the G6pase and PEPCK activity in the liver. Particularly, BCS extract (200 mg/kg) inhibited the HFD-induced increase in liver G6pase and PEPCK activity as compared to the MET_250_ (Table 9).

### 3.9. Lipid Metabolism-Related Gene Expression

#### 3.9.1. The mRNA Expressions in Liver Tissue

The HFD supply in the control group significantly (*p* < 0.01) increased the ACC1 mRNA expression and decreased the AMPKα1 and AMPKα2 mRNA expression in the liver tissue as compared to the NFD control group. However, all three doses of BCS extract (400, 200, and 100 mg/kg) significantly (*p* < 0.01) decreased the ACC1 mRNA expression and increased the AMPKα1 and AMPKα2 mRNA expressions in the liver. Particularly, the BCS (200 mg/kg) inhibited the HFD-induced increases in ACC1 mRNA expression and decreases in AMPKα1 and AMPKα2 mRNA expression in the liver as compared to the MET_250_ group (Table 10).

#### 3.9.2. The mRNA Expressions in Adipose Tissue

The HFD supply in the control group significantly (*p* < 0.01) increased the leptin, FAS, C/EBPa, C/EBPβ, PPARγ, and SREBP1c mRNA expression and decreased the PPARα, adiponectin, and UCP2 mRNA expression in adipose tissue as compared to the NFD control group. However, all three doses of BCS extract (400, 200, and 100 mg/kg) significantly (*p* < 0.01) reversed these expressions. Particularly, BCS (200 mg/kg) significantly inhibited the increased expressions of leptin, FAS, C/EBPa, C/EBPβ, PPARγ, and SREBP1c and decreased the expressions of PPARα, adiponectin, and UCP2 in HFD-induced adipose tissue, comparable to the MET_250_ group (Table 11).

## 4. Discussion

The incidence of metabolic syndrome and NAFLD is rapidly increasing worldwide [52,53]. Hepatic intracellular fat accumulation during NAFLD causes inflammation, liver damage, and fibrosis, which lead to more severe hepatocellular carcinoma, liver cirrhosis, and NASH [54,55]. However, there are no conclusive drugs for completely treating a metabolic syndrome [55,56]. Nevertheless, there are some drugs that can control metabolic syndrome, but their use is limited due to various side effects [57]. Therefore, changes in lifestyle, such as exercise therapy, are usually recommended for treating a metabolic syndrome [55]. Hence, an alternative drug with few adverse effects that can suppress the overall metabolic syndrome and can be used for a prolonged period of time needs to be developed. As black cumin is known for its glycemic control activity through its glycated pigment inhibitory effect [30], its potential as a preventive and therapeutic agent for diabetes is attracting attention [31,32]. Therefore, in the present study, we analyzed the pharmacological potential of BCS extract.

Fat accumulation is the most common phenomenon caused by obesity [7,8,9,10]. Changes in adipokine secretion by adipose tissue lead to complex diseases, such as obesity and insulin resistance [8,9,58]. All three doses of BCS extract (400, 200, and 100 mg/kg) significantly and dose-dependently inhibited the fat accumulation and adipocyte hypertrophy. Particularly, BCS extract (200 mg/kg) administration significantly inhibited the HFD-induced fat accumulation and adipocyte hypertrophy as compared to the metformin (250 mg/kg)-administered group. These results show that, at least under the studied circumstances, the oral administration of BCS extract (400, 200, and 100 mg/kg) showed a dose-dependent improvement trend in obesity as compared to the metformin (250 mg/kg). Similar to earlier studies [7,8,9,10], the feed intake in the HFD control group was significantly decreased as compared to the NFD control group. Nevertheless, from a caloric point of view, the effect on the induction of obesity was considered not significant, as the calorific value of HFD used in this study was 4.73 kcal/g, and that of NFD was 4.00 kcal/g, which is slightly less than the calorie value of HFD. Furthermore, no significant differences in feed intake were observed in any of the experimental groups. Thus, it is judged that the pharmacological properties of BCS extract observed in this study are not simply due to a reduction in feed intake.

The enzymes present in zymogen granules digest proteins and fats [59]. The pancreas causes a significant decrease in these zymogen granules, along with the accumulation of fat during obesity [7,8,9,10]. The HFD control group showed the promoted secretion of pancreatic enzymes involved in fat digestion, as a significant decrease in accumulated zymogen granules was observed histopathologically compared to the NFD control group. Accordingly, obesity was confirmed due to increased fat absorption. Furthermore, in histopathological examination, the proportion of zymogen granules in the pancreas was significantly inhibited by all three doses of BCS extract (400, 200, and 100 mg/kg). Particularly, BCS extract (200 mg/kg) administration inhibited the reduction in the HFD-induced zymogen granules ratio, comparable to that in the metformin (250 mg/kg)-administered group. These results show a dose-dependent improvement trend in HFD mice through BCS extract-inhibited fat absorption by regulating the secretion of enzymes involved in pancreatic fat digestion.

HbA1c is a key indicator for long-term hyperglycemia [60,61]. In type 2 diabetes, an increase in the blood insulin and HbA1c content and long-term hyperglycemia is generally observed [7,8,9,10,62]. In addition, a long-term supply of HFD in mice supports the homeostasis of blood sugar, allows for growth and the expansion of the pancreatic islet, and increases insulin- and glucagon-producing cells [63,64]. Similar to previous studies (64,65), the HFD control group in this study showed an increase in the number and expansion of pancreatic islets, along with significant increases in blood glucose, blood insulin, and HbA1c contents and increases in the glucagon, insulin, and insulin/glucagon ratio, as confirmed histopathologically. This shows the successful induction of typical insulin-resistant type 2 diabetes mellitus, whereas BCS extract (400, 200, and 100 mg/kg)-administered groups dose-dependently suppressed the blood sugar, blood insulin, and HbA1c content and histological and immunohistological changes in the pancreatic endocrine region. In particular, the inhibitory effect of HFD-induced insulin-resistant type 2 diabetes in BCS extract (200 mg/kg) was comparable to that of the metformin (250 mg/kg)-administered group. These results are judged as clear evidence showing a dose-dependent improvement effect on blood glucose through the control of pancreatic endocrine function by the oral administration of BCS extract.

In a persistent hyperglycemic state, hyperlipidemia results in several complications [11], such as a decrease in HDL along with an increase in LDL, TC, and TG content [7,8,9,10]. Therefore, the antihyperlipidemic effect of candidate substances is important to study [7,8,9,10]. All three doses of BCS extract (400, 200, and 100 mg/kg) showed significant decreases in the blood TG, TC, and LDL content and increases in the HDL content. In particular, the inhibitory effect of HFD-induced hyperlipidemia in the BCS extract (200 mg/kg)-administered group was comparable to that of the metformin (250 mg/kg)-administered group. In addition to the hyperlipidemia improvement effects, increases in the fecal TC and TG content and increases in the pancreatic zymogen content were observed histopathologically. Similar results were observed in the metformin (250 mg/kg)-supplied group. These improvement effects are judged to be due to the increase in lipid excretion caused by the suppression of lipids digestion by regulating the secretion of pancreatic digestive enzymes and a decrease in absorption.

ALT, AST, GGT, LDH, and ALP are the most common blood chemistry values for measuring liver damage [65]. Hepatic fat degradation and accumulation in the liver causes an increase in the blood ALT, AST, GGT, LDH, and ALP content [7,8,9,10]. HFD supply causes liver damage, accompanied by hyperlipidemia, an increase in the liver weight, and a consequent increase in the blood AST, ALT, ALP, LDH, and GGT content, thereby inducing NAFLD [7,8,9,10]. The BCS extract (400, 200, and 100 mg/kg) significantly and dose-dependently inhibited the HFD-induced increases in the absolute liver weight and blood ALT, AST, GGT, LDH, and ALP content. The histopathological examination showed a significant suppression of changes in hepatic fat and hepatocyte hypertrophy. In particular, the HFD-induced NAFLD improvement effect in BCS extract (200 mg/kg)-administered mice was similar to that of the metformin (250 mg/kg)-administered mice. It is judged as clear evidence that the BCS extract at a dose of 200 mg/kg shows improvement in the HFD-induced NAFLD, similar to that of the metformin (250 mg/kg)-administered group.

The blood creatinine and BUN contents are some of the most important blood chemistry values indicating the condition of kidneys [65]. The HFD supply in the control group showed significant increases in the blood creatinine and BUN content and the absolute kidney weights. Histopathological examinations confirmed tubular vacuolization, which is characterized by the infiltration of fat droplets. These findings of diabetic nephropathy were significantly inhibited by all three doses of BCS extract (400, 200, and 100 mg/kg). In particular, the administration of BCS extract (200 mg/kg) showed metformin (250 mg/kg)-comparable improvement effects for the HFD-induced diabetic nephropathy. These results show that the oral administration of BCS extract (400, 200, and 100 mg/kg) improves HFD-induced diabetic nephropathy as compared to that of metformin (250 mg/kg).

The free radicals play a significant role in the induction of diabetes and diabetic complications [66]. When diabetes develops, more free radicals are formed due to the increase in oxidative stress, along with the inhibition of endogenous antioxidants [67]. The free radicals formed in this way are the main cause of diabetic complications [67,68]. Similar to earlier studies [62,69], the HFD control group showed a decrease in the endogenous antioxidant (GSH) content, a decrease in the endogenous antioxidant enzymes activity (SOD and CAT), and an increase in the MDA content in the liver parenchyma by lipid peroxidation. All three doses of BCS extract (400, 200, and 100 mg/kg) significantly inhibited the disturbances in the antioxidant defense system and related lipid peroxidation. In particular, the antioxidant effects of the BCS extract (200 mg/kg) were comparable to those of the metformin (250 mg/kg). These results provide clear evidence that the oral administration of BCS extract (400, 200, and 100 mg/kg) shows significant antioxidant effects in HFD-fed mice as compared to the metformin (250 mg/kg).

Increases in PEPCK and G6pase enzyme activity and decreases in GK enzyme activity in the HFD-supplied experimental animal models have been associated with hyperglycemia [7,8,9,10,62]. Similar to previous studies [7,8,9,10], the HFD control group in this study showed significant decreases in GK enzyme activity and increases in the PEPCK and G6pase enzyme activities in the liver, compared to the NFD control group. However, changes in the GK, G6pase, and PEPCK activities, which are enzymes related to glucose metabolism in the liver tissue, were significantly inhibited by all three doses of BCS (400, 200, and 100 mg/kg). In particular, the oral administration of 200 mg/kg of BCS extract showed improvement effects for HFD-induced GK, G6pase, and PEPCK activities, comparable to those of the metformin (250 mg/kg). These findings show that supplying BCS extract (400, 200, and 100 mg/kg) to mice dose-dependently modulated the enzymes related to glucose metabolism, showing considerable effects at a dose of 200 mg/kg in HFD-fed mice.

To understand the mechanism of action of candidate substances regarding related complications including NAFLD, obesity, and diabetes, the mRNA expression of genes linked with the fat metabolism in the liver and adipose tissues was recorded. AMPK activity in the adipose and liver is considered one of the most important cell signaling pathways that regulate fat metabolism and blood sugar [70,71]. An increase in insulin sensitivity and fat oxidation by adipocytes-derived adiponectin occurs in an AMPK-dependent manner [8,9,14,72,73]. Therefore, the mRNA expressions of genes involved in the AMPK signaling pathway in the liver and adipose tissue were studied. The results in the HFD control group were directly related to the NAFLD pathogenesis. Decreased AMPKα1 and AMPKα2 mRNA expression and increased ACC1 mRNA expression were observed in liver tissue. Increased mRNA expression for C/EBPa, C/EBPβ, FAS, PPARγ, SREBP1c, and leptin and decreased mRNA expression for UCP2, adiponectin, and PPARα were observed in adipose tissue, whereas the HFD-induced changes in the AMPK and lipid metabolism-related gene expression were significantly inhibited by all three doses of BCS extract (400, 200, and 100 mg/kg). These results suggest that, at least under the circumstances of the present study, the oral supply of BCS extract (400, 200, and 100 mg/kg) can consistently improve lipid metabolism in a dose-dependent manner through the inhibition of lipid synthesis mediated by the regulation of AMPK expression and increased fatty acid oxidation in HFD-fed mice as compared to the metformin (250 mg/kg).

Even though the BCS extract showed a dose-dependent tendency for improving metabolic disorders, further studies checking the dose-dependent significance between different drug groups are required to verify the findings of this study. Rather than a purified extract, a crude BCS aqueous extract was used in this study. Thus, further studies could focus on composition analyses of the BCS super critical aqueous extract to identify the active substances and to study the efficacy relationship with thymoquinone, a known component in back cumin. In addition, there may be differences in efficacy depending on species specificity between humans and mice. Thus, the possibility of unexpected side effects cannot be ruled out.

## 5. Conclusions

The obesity, diabetes-related hyperlipidemia, NAFLD, and diabetic nephropathy improving effects of BCS extract (400, 200, and 100 mg/kg) were evaluated using a 45% Kcal HFD-fed mouse model. Continuous oral supplies of BCS extract (400, 200, and 100 mg/kg) for a period of 84 days significantly suppressed the diabetic complications in a dose-dependent manner. The inhibition of oxidative stress through lipid peroxidation, antioxidant activity, normalized activity of sugar metabolism-related enzymes in the liver, and normalized mRNA expressions of fat metabolism-related genes were noted in all BCS extract (400, 200, and 100 mg/kg)-supplied groups. Particularly, the administration of 200 mg/kg of the BCS extract significantly inhibited the HFD-induced diabetic nephropathy, hyperlipidemia, obesity, and NAFLD and showed comparable results to the metformin (250 mg/kg)-supplied group. The oral administration of BCS extract (400, 200, and 100 mg/kg), at least under the circumstances of the present study, consistently inhibited the insulin resistance by regulating the AMPK expression, type 2 obese diabetes, and the diabetic complications associated with oxidative stress, such as hyperlipidemia. The dose-dependent improvement effects for NAFLD and renal damage show an alleviating effect of BCS extract, particularly at a concentration of 200 mg/kg, which was comparable to that of the metformin (250 mg/kg)-administered group. Therefore, BCS aqueous extract, at a suitable dose, is expected to have a high potential for development as an effective treatment agent or functional food material for various diabetic complications including obesity, diabetes, and NAFLD.

## Figures and Tables

**Table 1:** Absolute and relative weights of fat pads in the HFD- and NFD-supplied groups.

Groups	Absolute Weights (g)		Relative Weights (% of Body Weights)	
	Periovarian Fat Pads	Abdominal Wall Fat Pads	Periovarian Fat Pads	Abdominal Wall Fat Pads
Control groups				
NFD	0.061 ± 0.025	0.051 ± 0.031	0.204 ± 0.085	0.171 ± 0.105
HFD	0.459 ± 0.114 ^c^	0.426 ± 0.099 ^c^	1.029 ± 0.262 ^a^	0.954 ± 0.227 ^a^
MET_250_	0.198 ± 0.043 ^c,d^	0.246 ± 0.031 ^c,d^	0.531 ± 0.101 ^a,c^	0.666 ± 0.094 ^a,d^
Test substance groups				
BCS_400_	0.152 ± 0.042 ^c,d^	0.168 ± 0.064 ^c,d^	0.437 ± 0.118 ^a,c^	0.483 ± 0.183 ^a,c^
BCS_200_	0.203 ± 0.030 ^c,d^	0.242 ± 0.040 ^c,d^	0.543 ± 0.080 ^a,c^	0.647 ± 0.107 ^a,d^
BCS_100_	0.283 ± 0.032 ^c,e^	0.285 ± 0.024 ^c,e^	0.733 ± 0.101 ^a^	0.735 ± 0.061 ^a^

Values are expressed as means ± S.D. (*n* = 10); NFD: 10 mL/kg of normal pellet diet orally administered mice; HFD: 10 mL/kg of 45% Kcal high-fat diet-supplied group; MET_250_: Metformin (250 mg/kg)-supplied group; BCS_400, 200, 100_: Black cumin seeds extract-administered groups at concentrations of 400, 200, and 100 mg/kg, respectively; DT3: Dunnett’s T3 test; THSD: Tukey’s Honest Significant Difference test; ^a^ *p* < 0.01 as compared to the NFD control group by the THSD test; ^c^ *p* < 0.01 as compared to the NFD control group by the DT3 test; ^d^ *p* < 0.01 and ^e^ *p* < 0.05 as compared to the HFD control group by the DT3 test.

**Table 2:** Histopathology–histomorphometry of abdominal and periovarian wall accumulated fat pads in HFD- and NFD-supplied groups.

Groups	Periovarian Fat Pads		Abdominal Wall Fat Pads	
	Thickness (mm)	Adipocyte Diameters (μm)	Thickness (mm)	Adipocyte Diameters (μm)
Control groups				
NFD	1.22 ± 0.29	31.91 ± 5.59	1.72 ± 0.65	44.45 ± 6.38
HFD	5.01 ± 0.89 ^c^	102.92 ± 14.89 ^a^	6.19 ± 0.99 ^a^	108.56 ± 11.38 ^a^
MET_250_	3.05 ± 0.42 ^c,d^	64.47 ± 11.15 ^a,b^	3.48 ± 0.52 ^a,b^	70.49 ± 13.42 ^a,b^
Test substance groups				
BCS_400_	2.48 ± 0.60 ^c,d^	43.91 ± 10.01 ^b^	2.71 ± 0.76 ^b^	58.44 ± 12.23 ^b^
BCS_200_	3.06 ± 0.50 ^c,d^	65.14 ± 11.66 ^a,b^	3.45 ± 0.72 ^a,b^	71.62 ± 11.42 ^a,b^
BCS_100_	3.54 ± 0.54 ^c,d^	78.17 ± 12.01 ^a,b^	4.19 ± 0.92 ^a,b^	83.00 ± 13.47 ^a,b^

DT3: Dunnett’s T3 test; THSD: Tukey’s Honest Significant Difference test; ^a^ *p* < 0.01 as compared to the NFD control group by the THSD test; ^b^ *p* < 0.01 as compared to the HFD control group by the THSD test; ^c^ *p* < 0.01 as compared to the NFD control group by the DT3 test; ^d^ *p* < 0.01 as compared to the HFD control group by the DT3 test.

**Table 3:** Histopathology–histomorphometry of the pancreas in HFD- and NFD-supplied groups.

Groups	Zymogen Granules (%/mm^2^ of Exocrine)	Mean Islet Numbers (Numbers/10 mm^2^)	Mean Islet Diameter (μm/islet)	Insulin-IR Cells (cells/mm^2^) [A]	Glucagon-IR Cells (cells/mm^2^) [B]	Insulin/Glucagon Ratio [A/B]
Control groups						
NFD	64.15 ± 11.16	5.30 ± 1.16	128.33 ± 14.32	171.40 ± 18.23	47.40 ± 5.42	3.63 ± 0.21
HFD	11.58 ± 2.37 ^a^	20.90 ± 2.33 ^a^	225.07 ± 18.93 ^a^	2931.00 ± 281.72 ^c^	454.50 ± 44.35 ^c^	6.45 ± 0.19 ^a^
MET_250_	40.13 ± 15.24 ^a,b^	12.20 ± 2.30 ^a,b^	169.96 ± 16.26 ^a,b^	1369.00 ± 331.80 ^c,d^	310.70 ± 46.28 ^c,d^	4.37 ± 0.49 ^a,b^
Test substance groups						
BCS_400_	50.69 ± 10.92 ^b^	8.80 ± 1.99 ^a,b^	146.50 ± 12.35 ^b^	785.50 ± 129.75 ^c,d^	212.30 ± 29.89 ^c,d^	3.72 ± 0.50 ^b^
BCS_200_	39.45 ± 10.86 ^a,b^	12.10 ± 2.42 ^a,b^	167.45 ± 14.37 ^a,b^	1292.90 ± 181.44 ^c,d^	301.70 ± 37.14 ^c,d^	4.29 ± 0.39 ^a,b^
BCS_100_	25.79 ± 10.31 ^a^	16.20 ± 1.03 ^a,b^	184.82 ± 13.28 ^a,b^	2061.70 ± 256.80 ^c,d^	359.20 ± 41.57 ^c,d^	5.74 ± 0.25 ^a,b^

DT3: Dunnett’s T3 test; THSD: Tukey’s Honest Significant Difference test; ^a^ *p* < 0.01 as compared to the NFD control group by the THSD test; ^b^ *p* < 0.01 as compared to the HFD control group by the THSD test; ^c^ *p* < 0.01 as compared to the NFD control group by the DT3 test; ^d^ *p* < 0.01 as compared to the HFD control group by the DT3 test.

**Table 4:** Blood glucose and serum lipid content in HFD- and NFD-supplied groups.

Groups	Glucose (mg/dL)	Total Cholesterol (mg/dL)	Triglyceride (mg/dL)	Low-Density Lipoprotein (mg/dL)	High-Density Lipoprotein (mg/dL)
Control groups					
NFD	93.10 ± 13.19	82.20 ± 18.91	83.80 ± 15.08	19.10 ± 4.41	85.00 ± 11.11
HFD	252.20 ± 29.19 ^a^	274.80 ± 30.01 ^a^	244.80 ± 25.55 ^c^	98.50 ± 13.99 ^c^	22.80 ± 10.40 ^a^
MET_250_	172.00 ± 20.61 ^a,b^	174.10 ± 27.92 ^a,b^	154.50 ± 28.38 ^c,d^	54.40 ± 10.89 ^c,d^	48.10 ± 14.50 ^a,b^
Test substance groups					
BCS_400_	144.20 ± 14.73 ^a,b^	134.50 ± 14.68 ^a,b^	118.20 ± 15.36 ^c,d^	39.30 ± 11.09 ^c,d^	61.00 ± 12.63 ^a,b^
BCS_200_	173.20 ± 12.02 ^a,b^	175.20 ± 16.88 ^a,b^	153.80 ± 17.47 ^c,d^	54.50 ± 14.40 ^c,d^	48.80 ± 12.66 ^a,b^
BCS_100_	195.80 ± 17.54 ^a,b^	200.60 ± 18.93 ^a,b^	189.40 ± 13.61 ^c,d^	65.10 ± 11.05 ^c,d^	44.10 ± 10.21 ^a,b^

DT3: Dunnett’s T3 test; THSD: Tukey’s Honest Significant Difference test; ^a^ *p* < 0.01 as compared to the NFD control group by the THSD test; ^b^ *p* < 0.01 as compared to the HFD control group by the THSD test; ^c^ *p* < 0.01 as compared to the NFD control group by the DT3 test; ^d^ *p* < 0.01 as compared to the HFD control group by the DT3 test.

**Table 5:** Absolute and relative organ weights in HFD- and NFD-supplied groups.

Groups	Absolute Organ Weights (g)			Relative Organ Weights (% of Body Weights)		
	Liver	Kidney	Pancreas	Liver	Kidney	Pancreas
Control groups						
NFD	1.196 ± 0.069	0.192 ± 0.020	0.240 ± 0.016	3.989 ± 0.309	0.640 ± 0.065	0.802 ± 0.083
HFD	1.832 ± 0.067 ^a^	0.300 ± 0.019 ^a^	0.251 ± 0.026	4.107 ± 0.331	0.671 ± 0.057	0.565 ± 0.087 ^a^
MET_250_	1.476 ± 0.096 ^a,b^	0.240 ± 0.015 ^a,b^	0.255 ± 0.021	3.991 ± 0.329	0.651 ± 0.059	0.690 ± 0.071 ^d^
Test substance groups						
BCS_400_	1.356 ± 0.093 ^a,b^	0.229 ± 0.010 ^a,b^	0.257 ± 0.019	3.900 ± 0.302	0.658 ± 0.046	0.740 ± 0.076 ^c^
BCS_200_	1.466 ± 0.082 ^a,b^	0.241 ± 0.009 ^a,b^	0.259 ± 0.011	3.926 ± 0.230	0.645 ± 0.036	0.692 ± 0.023 ^b,d^
BCS_100_	1.552 ± 0.101 ^a,b^	0.254 ± 0.011 ^a,b^	0.253 ± 0.015	4.008 ± 0.298	0.657 ± 0.038	0.654 ± 0.038 ^a^

DT3: Dunnett’s T3 test; THSD: Tukey’s Honest Significant Difference test; ^a^ *p* < 0.01 as compared to the NFD control group by the THSD test; ^b^ *p* < 0.01 as compared to the HFD control group by the THSD test; ^c^ *p* < 0.01 as compared to the NFD control group by the DT3 test; ^d^ *p* < 0.01 as compared to the HFD control group by the DT3 test.

**Table 6:** Serum AST, ALT, ALP, LDH, GGT, BUN, and creatine content in HFD- and NFD-supplied groups.

Groups	AST (IU/L)	ALT (IU/L)	ALP (IU/L)	LDH (×10 IU/L)	GGT (IU/L)	BUN (mg/dL)	Creatinine (mg/dL)
Control groups							
NFD	87.10 ± 14.90	45.80 ± 10.30	80.00 ± 11.20	71.69 ± 19.56	4.50 ± 1.65	31.80 ± 10.80	0.71 ± 0.18
HFD	212.40 ± 22.41 ^a^	165.60 ± 15.79 ^a^	239.30 ± 23.63 ^c^	439.15 ± 56.90 ^c^	19.30 ± 2.83 ^a^	135.40 ± 21.39 ^c^	2.18 ± 0.32 ^a^
MET_250_	147.40 ± 19.31 ^a,b^	96.70 ± 14.68 ^a,b^	154.30 ± 25.51 ^c,e^	205.57 ± 44.31 ^c,d^	11.70 ± 1.49 ^a,b^	79.90 ± 13.10 ^c,e^	1.37 ± 0.19 ^a,b^
Test substance groups							
BCS_400_	125.10 ± 21.71 ^a,b^	74.30 ± 13.90 ^a,b^	127.40 ± 34.33 ^d,e^	136.44 ± 31.85 ^c,d^	9.50 ± 0.97 ^a,b^	55.30 ± 12.04 ^c,e^	1.13 ± 0.15 ^a,b^
BCS_200_	145.70 ± 16.67 ^a,b^	96.30 ± 11.89 ^a,b^	153.10 ± 21.00 ^c,e^	202.49 ± 30.92 ^c,d^	11.60 ± 1.58 ^a,b^	80.50 ± 13.64 ^c,e^	1.38 ± 0.21 ^a,b^
BCS_100_	162.70 ± 12.23 ^a,b^	123.80 ± 11.53 ^a,b^	184.00 ± 10.22 ^c,e^	286.38 ± 50.13 ^c,d^	13.80 ± 1.14 ^a,b^	92.30 ± 10.34 ^c,e^	1.66 ± 0.17 ^a,b^

DT3: Dunnett’s T3 test; THSD: Tukey’s Honest Significant Difference test; ^a^ *p* < 0.01 as compared to the NFD control group by the THSD test; ^b^ *p* < 0.01 as compared to the HFD control group by the THSD test; ^c^ *p* < 0.01 and ^d^ *p* < 0.05 as compared to the NFD control group by the DT3 test; ^e^ *p* < 0.01 as compared to the HFD control group by the DT3 test.

**Table 7:** Histopathology–histomorphometry of the kidney and liver in the HFD- and NFD-supplied groups.

Groups	Liver Steatosis (%/mm^2^ of Hepatic Tissues)	Mean Hepatocyte Diameters (μm/cell)	Degenerative Renal Tubule Numbers (%)
Control groups			
NFD	3.82 ± 2.88	15.24 ± 1.89	6.50 ± 3.03
HFD	79.73 ± 10.31 ^a^	36.48 ± 4.03 ^a^	79.60 ± 10.74 ^c^
MET_250_	46.70 ± 10.68 ^a,b^	22.96 ± 3.15 ^a,b^	41.80 ± 10.97 ^c,d^
Test substance groups			
BCS_400_	33.73 ± 11.21 ^a,b^	18.77 ± 3.02 ^b^	27.50 ± 10.27 ^c,d^
BCS_200_	45.61 ± 11.16 ^a,b^	23.26 ± 3.83 ^a,b^	42.90 ± 10.83 ^c,d^
BCS_100_	55.09 ± 10.31 ^a,b^	25.92 ± 2.45 ^a,b^	56.60 ± 10.85 ^c,d^

DT3: Dunnett’s T3 test; THSD: Tukey’s Honest Significant Difference test; ^a^ *p* < 0.01 as compared to the NFD control group by the THSD test; ^b^ *p* < 0.01 as compared to the HFD control group by the THSD test; ^c^ *p* < 0.01 as compared to the NFD control group by the DT3 test; ^d^ *p* < 0.01 as compared to the HFD control group by the DT3 test.

**Table 8:** Antioxidant defense systems and liver lipid peroxidation in HFD- and NFD-supplied groups.

Groups	Lipid Peroxidation	Antioxidant Defense System		
	Malondialdehyde (nM/mg Tissue)	Glutathione (μM/mg Tissue)	Catalase (U/mg Tissue)	SOD (U/mg Tissue)
Control groups				
NFD	9.96 ± 3.52	77.47 ± 18.36	78.27 ± 13.10	7.91 ± 1.98
HFD	89.02 ± 12.13 ^a^	11.72 ± 2.65 ^c^	11.13 ± 3.10 ^a^	0.91 ± 0.30 ^c^
MET_250_	43.71 ± 11.01 ^a,b^	31.19 ± 11.93 ^c,e^	30.89 ± 11.89 ^a,b^	2.61 ± 1.17 ^c,f^
Test substance groups				
BCS_400_	33.15 ± 10.28 ^a,b^	49.24 ± 14.81 ^d,e^	46.78 ± 14.82 ^a,b^	4.01 ± 1.73 ^c,e^
BCS_200_	44.76 ± 10.41 ^a,b^	31.98 ± 12.86 ^c,e^	31.36 ± 10.81 ^a,b^	2.62 ± 0.84 ^c,e^
BCS_100_	61.75 ± 12.20 ^a,b^	24.05 ± 10.24 ^c,f^	24.01 ± 10.58 ^a^	2.07 ± 0.87 ^c,f^

DT3: Dunnett’s T3 test; THSD: Tukey’s Honest Significant Difference test; ^a^ *p* < 0.01 as compared to the NFD control group by the THSD test; ^b^ *p* < 0.01 as compared to the HFD control group by the THSD test; ^c^ *p* < 0.01 and ^d^ *p* < 0.05 as compared to the NFD control group by the DT3 test; ^e^ *p* < 0.01 and ^f^ *p* < 0.05 as compared to the HFD control group by the DT3 test.

**Table 9:** Activities of hepatic glucose-regulating enzymes in the HFD- and NFD-supplied groups.

Groups	Glucokinase (nM/min/mg Protein)	Glucose-6-Phosphatase (nM/min/mg Protein)	PEPCK (nM/min/mg Protein)
Control groups			
NFD	6.60 ± 1.35	114.68 ± 27.91	1.57 ± 0.68
HFD	1.61 ± 0.42 ^c^	364.56 ± 107.72 ^c^	5.92 ± 1.20 ^a^
MET_250_	3.72 ± 1.44 ^c,e^	194.23 ± 33.24 ^c,d^	3.39 ± 0.55 ^a,b^
Test substance groups			
BCS_400_	4.26 ± 0.97 ^c,d^	152.08 ± 21.94 ^d^	2.51 ± 0.40 ^b^
BCS_200_	3.74 ± 0.61 ^c,d^	194.03 ± 27.30 ^c,d^	3.38 ± 0.68 ^a,b^
BCS_100_	2.83 ± 0.74 ^c,d^	231.11 ± 24.75 ^c,e^	4.07 ± 0.74 ^a,b^

DT3: Dunnett’s T3 test; THSD: Tukey’s Honest Significant Difference test; ^a^ *p* < 0.01 as compared to the NFD control group by the THSD test; ^b^ *p* < 0.01 as compared to the HFD control group by the THSD test; ^c^ *p* < 0.01 as compared to the NFD control group by the DT3 test; ^d^ *p* < 0.01 and ^e^ *p* < 0.05 as compared to the HFD control group by the DT3 test.

**Table 10:** The mRNA expressions of genes involved in liver lipid metabolism in the HFD- and NFD-supplied groups.

Groups	Hepatic Tissue (Relative to Control—GAPDH)		
	ACC1	AMPKα1	AMPKα2
Control groups			
NFD	1.01 ± 0.07	1.00 ± 0.06	1.00 ± 0.07
HFD	5.50 ± 0.79 ^a^	0.24 ± 0.04 ^a^	0.23 ± 0.05 ^a^
MET_250_	2.99 ± 0.65 ^a,b^	0.52 ± 0.11 ^a,b^	0.43 ± 0.13 ^a,b^
Test substance groups			
BCS_400_	2.16 ± 0.53 ^a,b^	0.67 ± 0.17 ^a,b^	0.63 ± 0.16 ^a,b^
BCS_200_	2.94 ± 0.65 ^a,b^	0.52 ± 0.11 ^a,b^	0.44 ± 0.06 ^a,b^
BCS_100_	3.56 ± 0.76 ^a,b^	0.43 ± 0.11 ^a,b^	0.39 ± 0.08 ^a,b^

DT3: Dunnett’s T3 test; ^a^ *p* < 0.01 as compared to the NFD control group by the DT3 test; ^b^ *p* < 0.01 as compared to the HFD control group by the DT3 test.

**Table 11:** The mRNA expressions of lipid metabolism-related genes in adipose tissue of the HFD- and NFD-supplied groups.

Groups	Control		Reference	Test Substance Groups		
	NFD	HFD	MET_250_	BCS_400_	BCS_200_	BCS_100_
Adipose tissue (Relative to control—GAPDH)						
Leptin	1.00 ± 0.08	8.64 ± 1.62 ^c^	4.53 ± 1.20 ^c,d^	2.95 ± 1.15 ^c,d^	4.54 ± 1.04 ^c,d^	5.45 ± 1.22 ^c,d^
UCP2	1.01 ± 0.05	0.25 ± 0.05 ^a^	0.48 ± 0.11 ^a,b^	0.61 ± 0.13 ^a,b^	0.48 ± 0.11 ^a,b^	0.42 ± 0.08 ^a,b^
Adiponectin	1.00 ± 0.07	0.16 ± 0.03 ^c^	0.35 ± 0.08 ^c,d^	0.48 ± 0.16 ^c,d^	0.35 ± 0.10 ^c,d^	0.29 ± 0.08 ^c,d^
C/EBPα	1.01 ± 0.07	3.57 ± 0.80 ^c^	1.94 ± 0.38 ^c,d^	1.49 ± 0.21 ^c,d^	1.94 ± 0.33 ^c,d^	2.27 ± 0.30 ^c,d^
C/EBPβ	1.00 ± 0.05	4.15 ± 0.60 ^c^	2.45 ± 0.56 ^c,d^	1.79 ± 0.42 ^c,d^	2.44 ± 0.51 ^c,d^	2.89 ± 0.39 ^c,d^
SREBP1c	1.00 ± 0.07	3.15 ± 0.44 ^c^	1.95 ± 0.31 ^c,d^	1.41 ± 0.18 ^c,d^	1.94 ± 0.34 ^c,d^	2.30 ± 0.23 ^c,d^
PPARα	1.00 ± 0.07	0.18 ± 0.04 ^c^	0.40 ± 0.11 ^c,d^	0.53 ± 0.17 ^c,d^	0.40 ± 0.08 ^c,d^	0.33 ± 0.09 ^c,d^
PPARγ	1.00 ± 0.08	7.78 ± 0.87 ^c^	4.31 ± 0.94 ^c,d^	3.18 ± 0.68 ^c,d^	4.33 ± 0.74 ^c,d^	5.18 ± 1.32 ^c,d^
FAS	1.00 ± 0.08	17.08 ± 2.40 ^c^	10.01 ± 1.61 ^c,d^	7.12 ± 2.90 ^c,d^	10.00 ± 3.26 ^c,d^	11.68 ± 2.37 ^c,d^

DT3: Dunnett’s T3 test; THSD: Tukey’s Honest Significant Difference test; ^a^ *p* < 0.01 as compared to the NFD control group by the THSD test; ^b^ *p* < 0.01 as compared to the HFD control group by the THSD test; ^c^ *p* < 0.01 as compared to the NFD control group by the DT3 test; ^d^ *p* < 0.01 as compared to the HFD control group by the DT3 test.

## Data Availability

Not applicable.

## Supplementary Materials

The following supporting information can be downloaded at: https://www.mdpi.com/article/10.3390/metabo13040501/s1, Table S1: Formulas of normal and high-fat diets used in this study; Table S2: Oligonucleotides for real-time RT-PCR; Table S3. Changes in body weight and mean daily food consumption in NFD- or HFD-supplied mice; Figure S1. HPLC analysis of standard thymoquinone (A) and thymoquinone in the BSC extract (B). Figure S2. Representative gross body mass and abdominal fat pads with whole body DEXA images taken from NFD- or HFD-supplied mice; Figure S3. Representative histological images of the adipocytes, taken from NFD- or HFD-supplied mice periovarian and abdominal wall deposited fat pads; Figure S4. Representative general histological images of the pancreas, taken from NFD- or HFD-supplied mice; Figure S5. Representative histological images of the insulin- and glucagon-immunoreactive cells in the pancreas, taken from NFD- or HFD-supplied mice; Figure S6. Representative histological images of the liver, taken from NFD- or HFD-supplied mice; Figure S7. Representative histological images of the kidney, taken from NFD- or HFD-supplied mice.
