# Supplementary material for: Efficacy Confirmation Test of Black Cumin (Nigella sativa L.) Seeds Extract Using a High-Fat Diet Mouse Model
Source: Metabolites. 2023 Mar 30;13(4):501. doi: 10.3390/metabo13040501 (PMC10142846; doi:10.3390/metabo13040501)
Supplement: Supplementary file 1 [file metabolites-13-00501-s001.zip › metabolites-2302242-supplementary.pdf]

metabolites

MDPI

Supplementary Material

Efficacy Confirmation Test of Black Cumin (*Nigella sativa* L.) Seeds Extract Using High-Fat Diet Mouse Model

Khawaja Muhammad Imran Bashir [1,†], Joo Wan Kim [2,†], Jong-Kyu Kim [3], Yoon-Seok Chun [3], Jae-Suk Choi [4,*], and Sae-Kwang Ku [5,*]

1 German Engineering Research Center for Life Science Technologies in Medicine and Environment, Busan 46742, Republic of Korea; imran.bashir@lstme.org
2 Department of Companion Animal Health, Daegu Haany University, Gyeongsan 38610, Republic of Korea; dvm_jkkim@dhu.ac.kr
3 AriBnC, Ltd., Yongin 16985, Republic of Korea; jkkim@aribnc.com (J.-K.K.); ceochun@aribnc.com (Y.S.C.)
4 Department of Seafood Science and Technology, The Institute of Marine Industry, Gyeongsang National University, Tongyeong 53064, Republic of Korea
5 Department of Anatomy and Histology, College of Korean Medicine, Daegu Haany University, Gyeongsan 38610, Republic of Korea
* Correspondence: jsc1008@gnu.ac.kr (J.-S.C); gucci200@dhu.ac.kr (S.-K.K.); Tel.: +82-55-772-9142 (J.-S.C.); +82-53-819-1549 (S.-K.K)
† These authors contributed equally to this work.

Table S1. Formulas of normal and high fat diets used in this study.

Compositions*	Normal pellet diet	High fat diet
Ingredient (g/kg)		
Sucrose	500	172.8
Casein	200	200
Corn starch	150	72.8
Cellulose	50	50
Soybean Oil	50	25
L-Cystein	3	3
Lard	0	177.5
Mineral mixture	35	35
Vitamin mixture	10	10
Choline bitartrate	2	2
Carbohydrate (% kcal)	64	35
Protein (% kcal)	20	20
Fat (% kcal)	16	45
Energy (kcal/g)	4.00	4.73

*45%Kcal Fat pellet diets [Research Diet, New Brunswick, NJ, USA] was used as high fat diet and normal rodents pellet diet [Purinafeed, Seungnam, Korea] was used as normal fat pellet diets

Table S2. Oligonucleotides for real time RT-PCR.

Target	5′ – 3′	Sequence	GenBank Accession Number
PPARα	Sense	ATGCCAGTACTGCCGTTTTC	NM_011144
	Antisense	GGCCTTGACCTTGTTCATGT	
PPARγ	Sense	AGTGGAGACCGCCCAGG	NM_001127330
	Antisense	GCAGCAGGTTGTCTTGGATGT	
Leptin	Sense	CCAAAACCCTCATCAAGACC	NM_008493
	Antisense	GTCCAACTGTTGAAGAATGTCCC	
UCP2	Sense	CCGCATTGGCCTCTACGACTCT	NM_011671
	Antisense	CCCCGAAGGCAGAAGTGAAGTG	
Adiponectin	Sense	CCCAAGGGAACTTGTGCAGGTTGGATG	NM_009605
	Antisense	GTTGGTATCATGGTAGAGAAGAAAGCC	
C/EBPα	Sense	TGGACAAGAACAGCAACGAGTAC	NM_001287523
	Antisense	CGGTCATTGTCACTGGTCAACT	
C/EBPβ	Sense	AAGCTGAGCGACGAGTACAAGA	NM_001287739
	Antisense	GTCAGCTCCAGCACCTTGTG	
SREBP1c	Sense	AGCCTGGCCATCTGTGAGAA	XM_006532714
	Antisense	CAGACTGGTACGGGCCACAA	
FAS	Sense	GCTGCGGAAACTTCAGGAAAT	NM_007988
	Antisense	AGAGACGTGTCACTCCTGGACTT	
ACC1	Sense	GCCATTGGTATTGGGGCTTAC	NM_133360
	Antisense	CCCGACCAAGGACTTTGTTG	
AMPKα1	Sense	AAGCCGACCCAATGACATCA	XM_011245321
	Antisense	CTTCCTTCGTACACGCAAAT	
AMPKα2	Sense	GATGATGAGGTGGTGGA	NM_178143
	Antisense	GCCGAGGACAAAGTGC	
GAPDH	Sense	CATCTTCCAGGAGCGAGACC	NM_008084
	Antisense	TCCACCACCCTGTTGCTGTA	

Real time RT-PCR = real-time reverse transcription polymerase chain reaction; PPAR = Peroxisome proliferator-activated receptor; UCP = Mitochondrial uncoupling protein; C/ EBP = CCAAT-enhancer-binding protein; SREBP = Sterol regulatory element-binding protein; ACC1 = Acetyl-CoA carboxylase 1; FAS = Fatty acid synthase; AMPK = 5' adenosine monophosphate-activated protein kinase; GAPDH = Glyceraldehyde 3-phosphate dehydrogenase

Table S3. Changes in body weight and mean daily food consumption in NFD or HFD supplied mice.

Groups	Body weights (g) at days after initial test substance treatment				Body weight gains during		Mean Daily Food Consumption (g)
	8 days before [A]	1 day before [B]	0 day* [C]	84 days* [D]	Adapt period [B-A]	Administration period [D-C]	
Control groups							
NFD	27.98 ± 2.00	28.99 ± 1.58	26.06 ± 1.56	30.07 ± 1.94	1.01 ± 0.87	4.01 ± 1.41	4.85 ± 0.72
HFD	27.93 ± 1.28	31.66 ± 1.88[a]	28.49 ± 1.81[a]	44.81 ± 3.07[c]	3.73 ± 1.09[a]	16.32 ± 2.10[a]	4.27 ± 0.24[c]
MET_{250}	27.96 ± 1.80	31.68 ± 1.88[a]	28.47 ± 1.48[a]	37.06 ± 1.87[ce]	3.72 ± 0.88[a]	8.59 ± 1.21[ab]	4.30 ± 0.50[d]
Test substance groups							
BCS_{400}	27.90 ± 0.85	31.68 ± 1.09[a]	28.65 ± 1.05[a]	34.80 ± 1.19[ce]	3.78 ± 1.10[a]	6.15 ± 0.96[ab]	4.29 ± 0.18[c]
BCS_{200}	27.92 ± 0.58	31.70 ± 1.02[a]	28.75 ± 0.89[a]	37.38 ± 1.40[ce]	3.78 ± 0.84[a]	8.63 ± 0.86[ab]	4.28 ± 0.23[c]
BCS_{100}	27.91 ± 0.54	31.68 ± 1.02[a]	28.81 ± 1.04[a]	38.76 ± 1.39[ce]	3.77 ± 1.01[a]	9.95 ± 1.16[ab]	4.30 ± 0.21[d]

Values are expressed as means ± S.D. ($n = 10$); NFD = Vehicle (distilled water) 10 mL/kg of normal pellet diet orally administered mice; HFD = Vehicle (distilled water) 10 mL/kg of 45% Kcal high fat diet orally administered mice; MET_{250} = Metformin administrated mice at a dose of 250 mg/kg; $BCS_{400, 200, 100}$ = Black cumin (*Nigella sativa* L.) seeds extract administered mice at doses of 400, 200, and 100 mg/kg; THSD = Tukey's Honest Significant Difference; DT3 = Dunnett's T3; *All animals were overnight fasted; [a] $p < 0.01$ as compared with NFD control by THSD test; [c] $p < 0.01$ and [d] $p < 0.05$ as compared with NFD control by DT3 test; [b] $p < 0.01$ as compared with HFD control by THSD test; [e] $p < 0.01$ as compared with HFD control by DT3 test

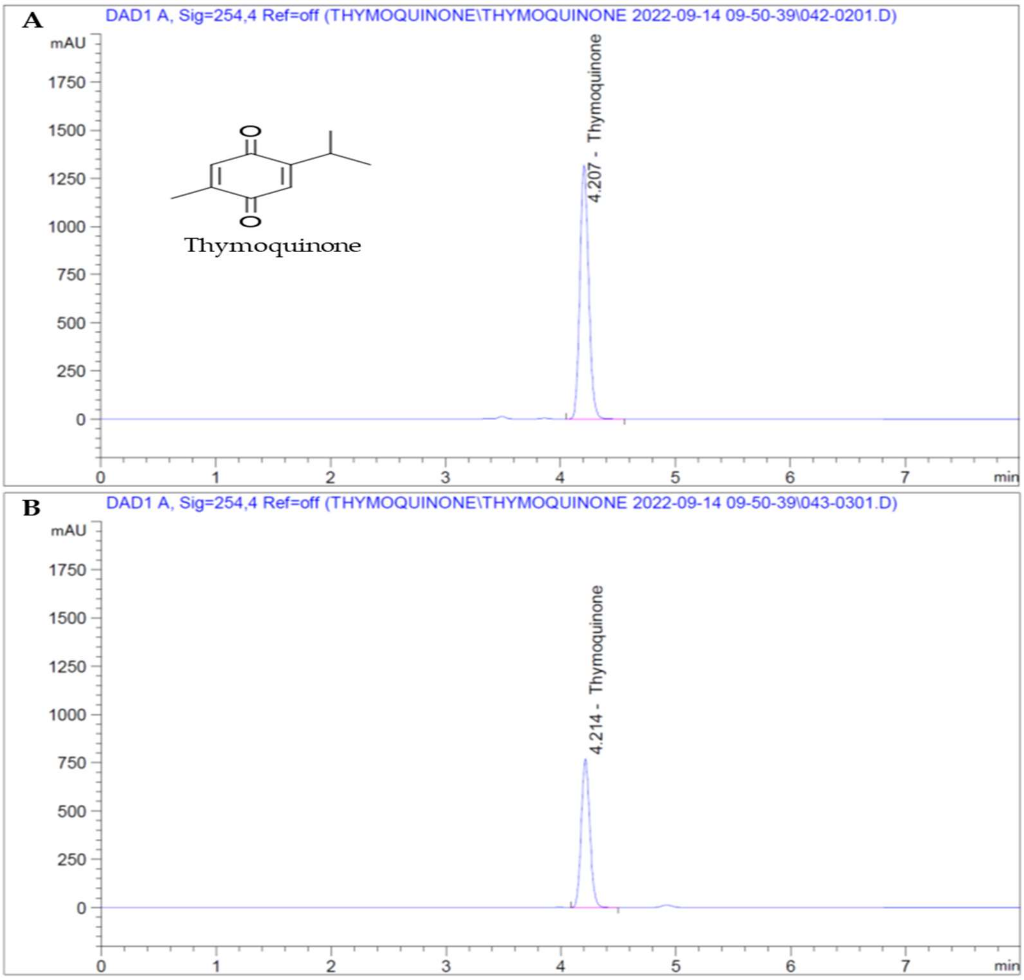

Figure S1. HPLC analysis of standard thymoquinone (**A**) and thymoquinone in the BSC extract **(B)**.

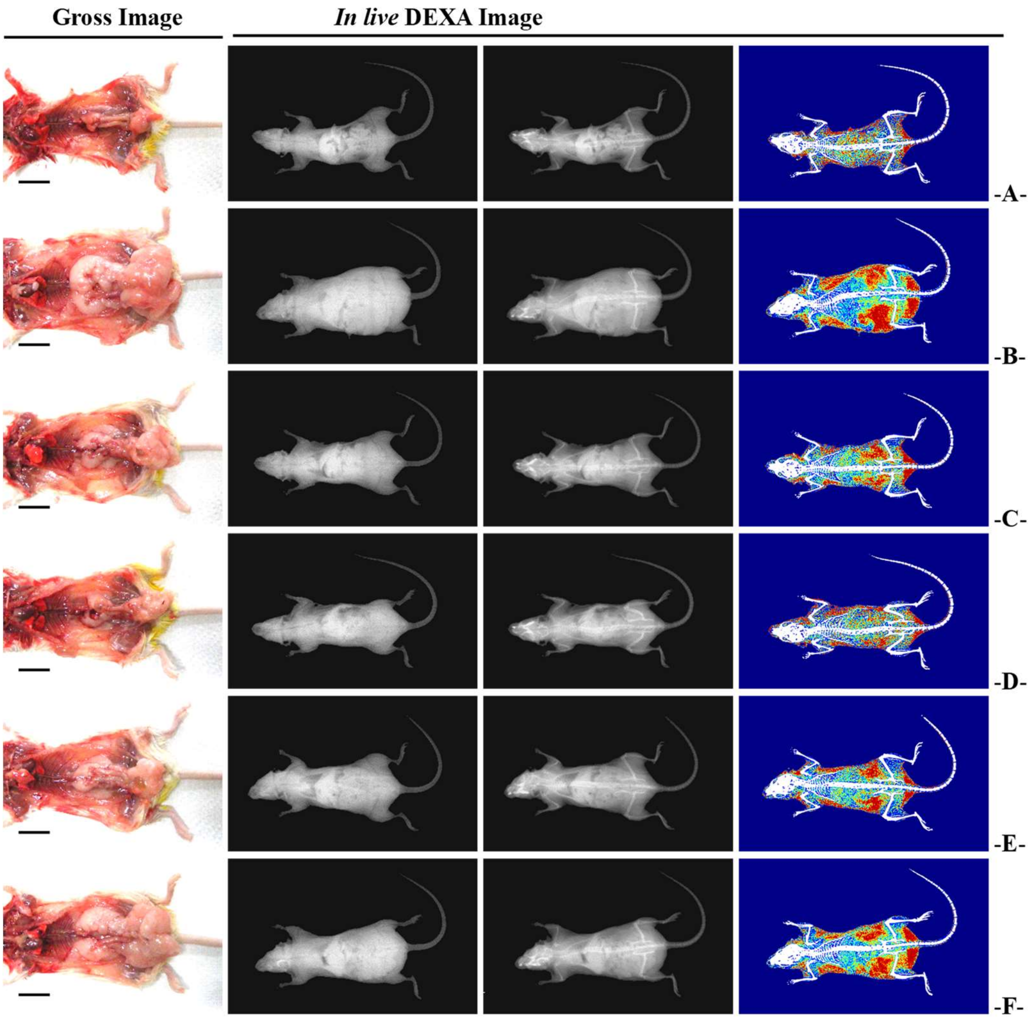

Figure S2. Representative gross body mass and abdominal fat pads with whole body DEXA images taken from NFD or HFD supplied mice.

A = Vehicle (distilled water) 10 mL/kg orally administered mice with NFD supply (NFD control); **B** = Vehicle 10 mL/kg orally administered mice with HFD supply (HFD control); **C** = Metformin (250 mg/kg) oral administered mice with HFD supply (MET_{250}); **D** = BCS (400 mg/kg) orally administered mice with HFD supply (BCS_{400}); **E** = BCS (200 mg/kg) orally administered mice with HFD supply (BCS_{200}); **F** = BCS (100 mg/kg) orally administered mice with HFD supply (BCS_{100}); NFD = Normal pellet diet; HFD = 45% Kcal high fat diet; BCS = Black cumin (*Nigella sativa* L.) seeds extract; DEXA = Dual-energy x-ray absorptiometry; Scale bar = 15.00 mm

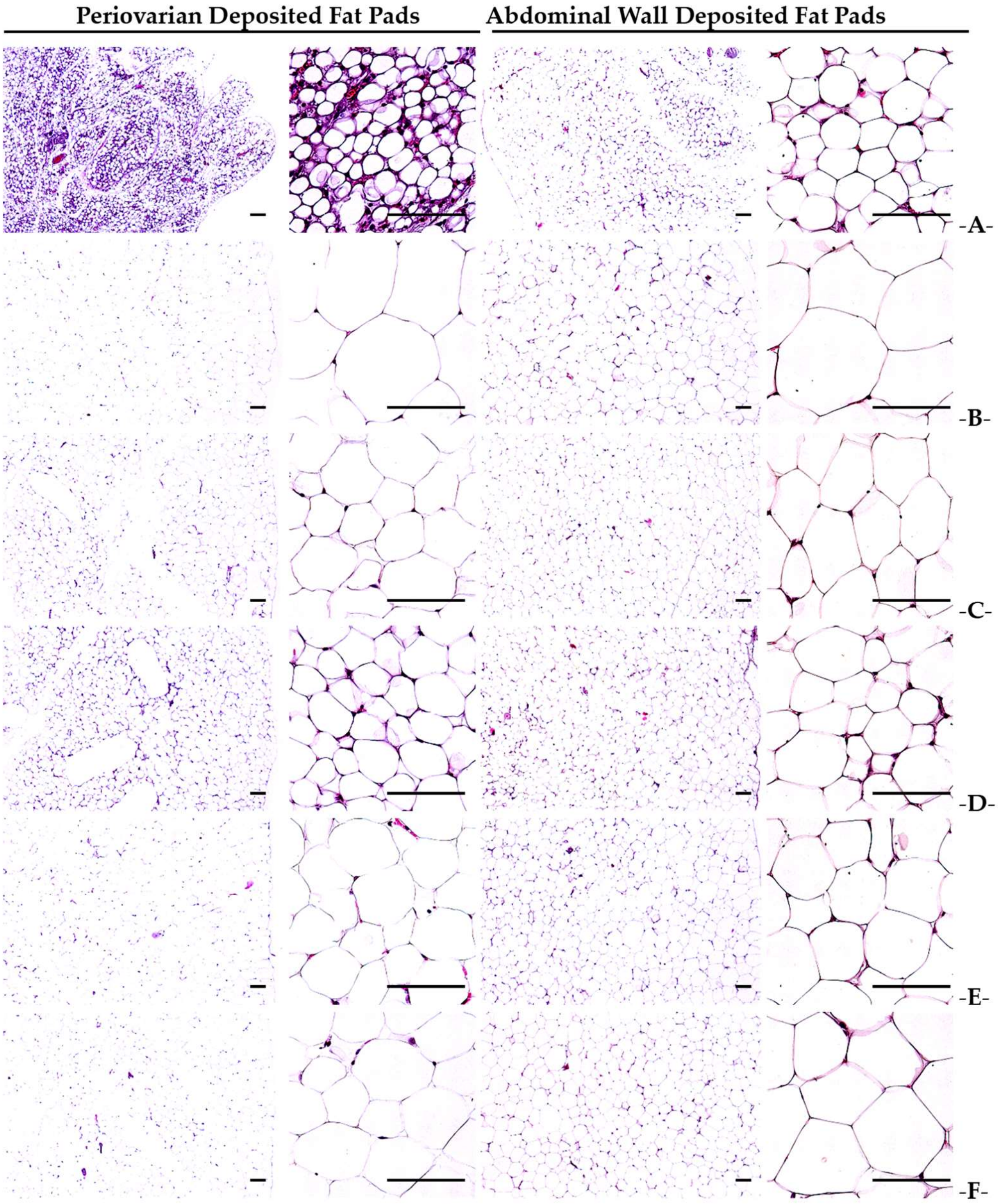

Figure S3. Representative histological images of the adipocytes, taken from NFD or HFD supplied mice periovarian and abdominal wall deposited fat pads.

A = Vehicle (distilled water) 10 mL/kg orally administered mice with NFD supply (NFD control); **B** = Vehicle 10 mL/kg orally administered mice with HFD supply (HFD control); **C** = Metformin (250 mg/kg) oral administered mice with HFD supply (MET_{250}); **D** = BCS (400 mg/kg) orally administered mice with HFD supply (BCS_{400}); **E** = BCS (200 mg/kg) orally administered mice with HFD supply (BCS_{200}); **F** = BCS (100 mg/kg) orally administered mice with HFD supply (BCS_{100}); NFD = Normal pellet diet; HFD = 45% Kcal high fat diet; BCS = Black cumin (*Nigella sativa* L.) seeds extract; All Hematoxylin and Eosin stain; Scale bars = 80 μm.

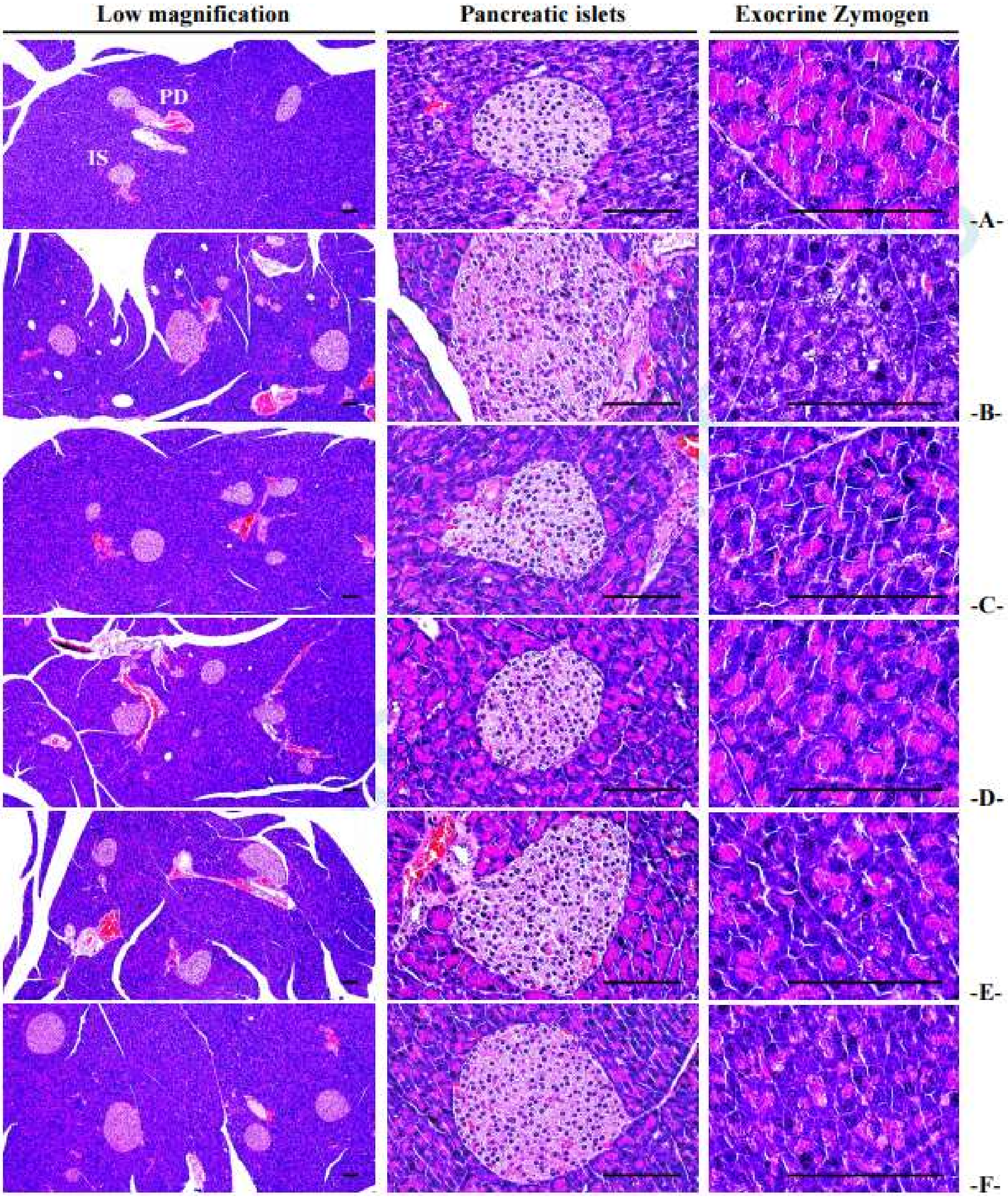

Figure S4. Representative general histological images of the pancreas, taken from NFD or HFD supplied mice.

A = Vehicle (distilled water) 10 mL/kg orally administered mice with NFD supply (NFD control); **B** = Vehicle 10 mL/kg orally administered mice with HFD supply (HFD control); **C** = Metformin (250 mg/kg) oral administered mice with HFD supply (MET_{250}); D = BCS (400 mg/kg) orally administered mice with HFD supply (BCS_{400}); E = BCS (200 mg/kg) orally administered mice with HFD supply (BCS_{200}); F = BCS (100 mg/kg) orally administered mice with HFD supply (BCS_{100}); NFD = Normal pellet diet; HFD = 45% Kcal high fat diet; BCS = Black cumin (*Nigella sativa* L.) seeds extract; IS = Pancreatic islet; PD = Pancreatic secretory duct; All Hematoxylin and Eosin stain; Scale bars = 80 μm

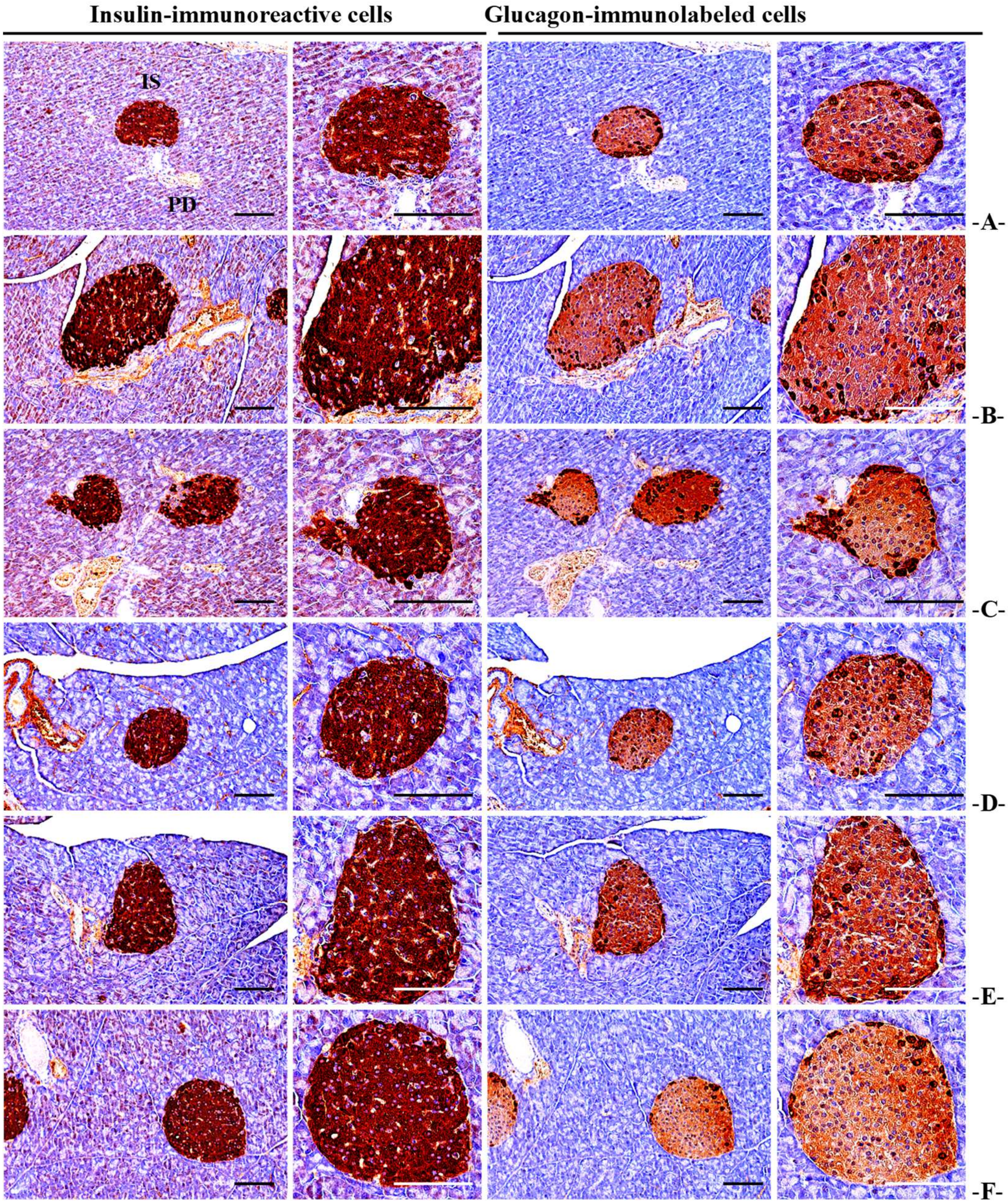

Figure S5. Representative histological images of the insulin- and glucagon- immunoreactive cells in the pancreas, taken from NFD or HFD supplied mice.

A = Vehicle (distilled water) 10 mL/kg orally administered mice with NFD supply (NFD control); **B** = Vehicle 10 mL/kg orally administered mice with HFD supply (HFD control); **C** = Metformin (250 mg/kg) oral administered mice with HFD supply (MET_{250}); **D** = BCS (400 mg/kg) orally administered mice with HFD supply (BCS_{400}); **E** = BCS (200 mg/kg) orally administered mice with HFD supply (BCS_{200}); **F** = BCS (100 mg/kg) orally administered mice with HFD supply (BCS_{100}); NFD = Normal pellet diet; HFD = 45% Kcal high fat diet; BCS = Black cumin (*Nigella sativa* L.) seeds extract; IS = Pancreatic islet; PD = Pancreatic duct; All immune-stained by avidin-biotin-peroxidase complex; Scale bars = 80 μm.

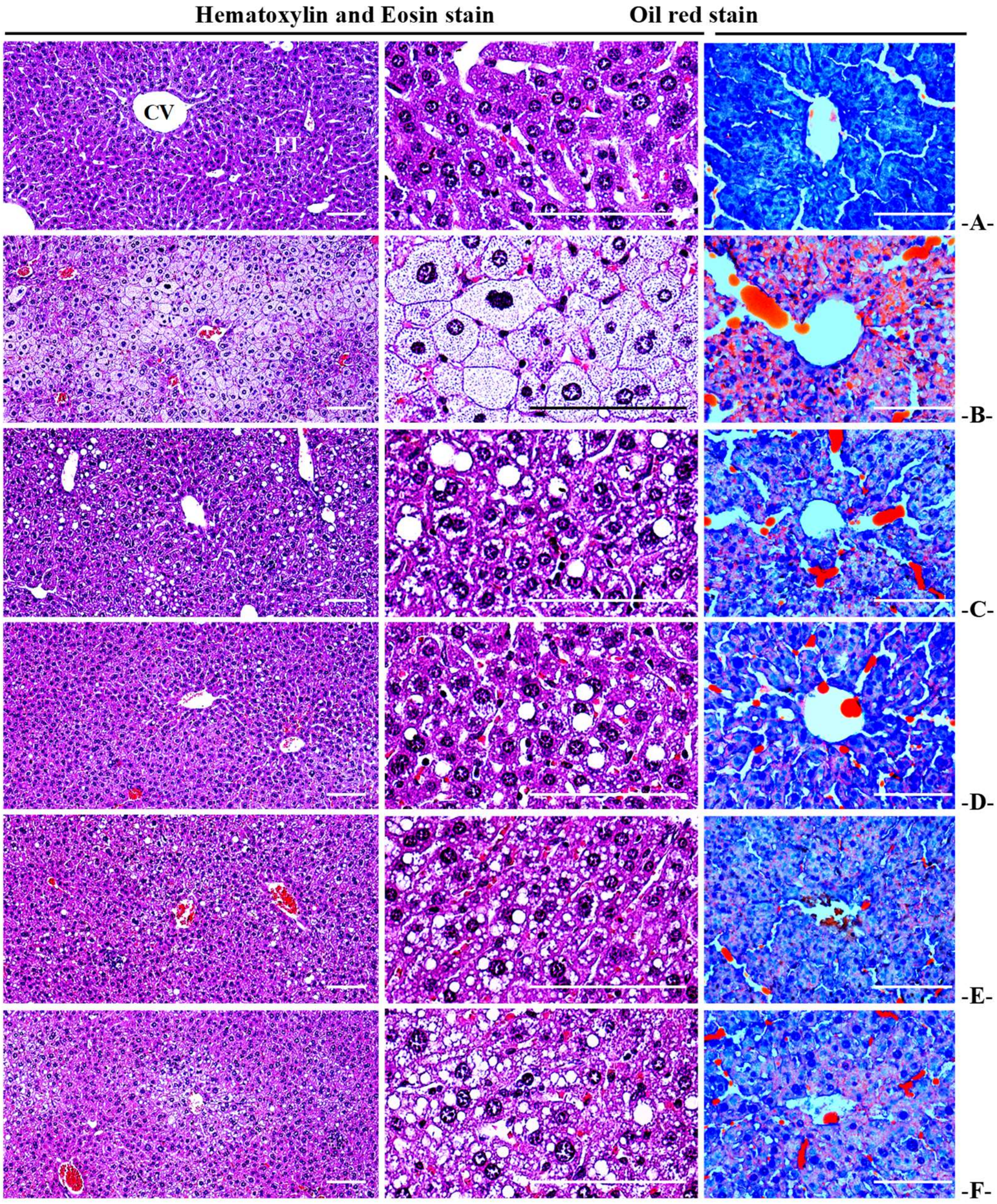

Figure S6. Representative histological images of the liver, taken from NFD or HFD supplied mice.

A = Vehicle (distilled water) 10 mL/kg orally administered mice with NFD supply (NFD control); **B** = Vehicle 10 mL/kg orally administered mice with HFD supply (HFD control); **C** = Metformin (250 mg/kg) oral administered mice with HFD supply (MET_{250}); **D** = BCS (400 mg/kg) orally administered mice with HFD supply (BCS_{400}); **E** = BCS (200 mg/kg) orally administered mice with HFD supply (BCS_{200}); **F** = BCS (100 mg/kg) orally administered mice with HFD supply (BCS_{100}); NFD = Normal pellet diet; HFD = 45% Kcal high fat diet; BCS = Black cumin (*Nigella sativa* L.) seeds extract; CV = Central vein; PT = Portal triad; Scale bars = 80 μm

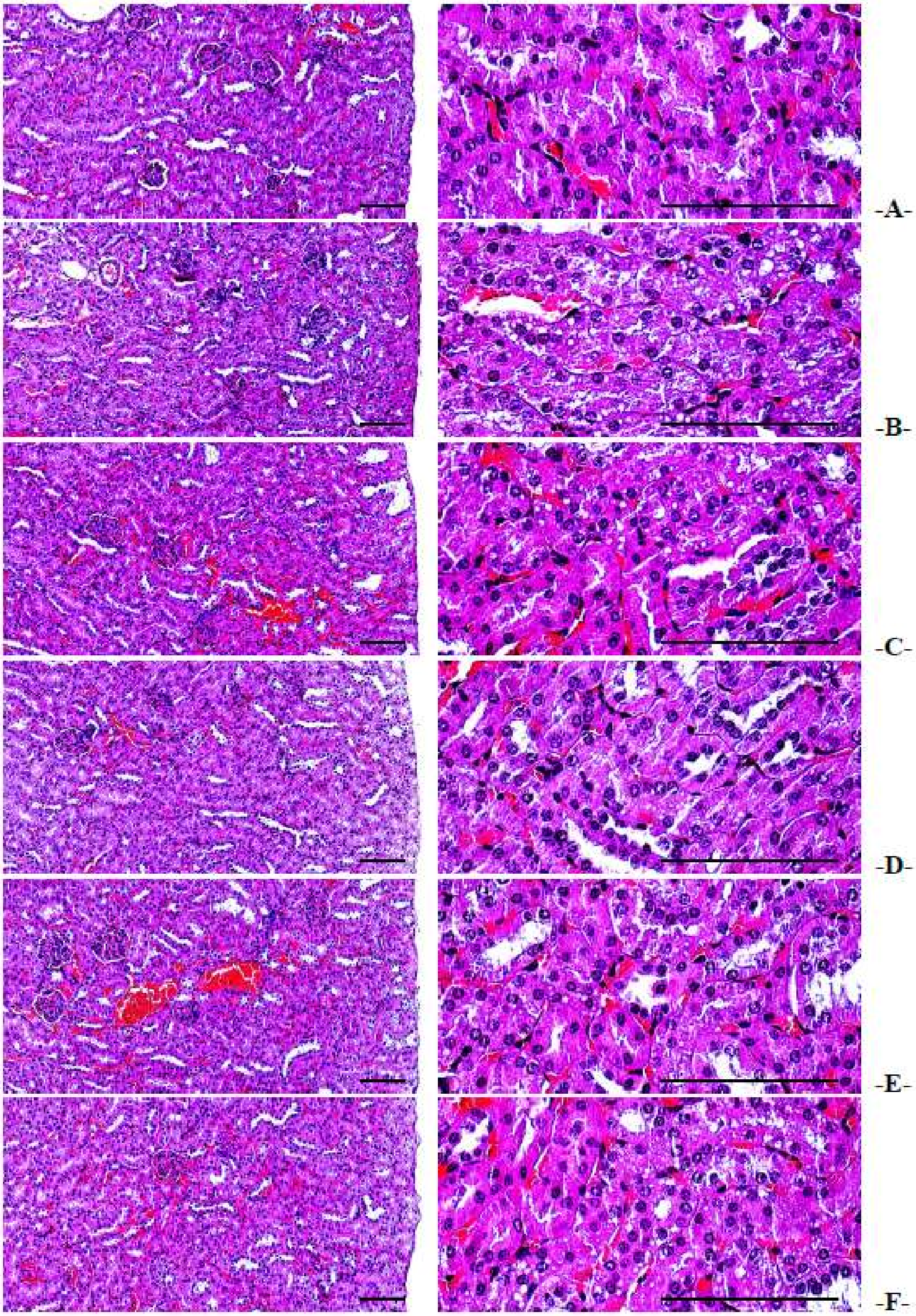

Figure S7. Representative histological images of the kidney, taken from NFD or HFD supplied mice.

A = Vehicle (distilled water) 10 mL/kg orally administered mice with NFD supply (NFD control); **B** = Vehicle 10 mL/kg orally administered mice with HFD supply (HFD control); **C** = Metformin (250 mg/kg) oral administered mice with HFD supply (MET_{250}); **D** = BCS (400 mg/kg) orally administered mice with HFD supply (BCS_{400}); **E** = BCS (200 mg/kg) orally administered mice with HFD supply (BCS_{200}); **F** = BCS (100 mg/kg) orally administered mice with HFD supply (BCS_{100}); NFD = Normal pellet diet; HFD = 45% Kcal high fat diet; BCS = Black cumin (*Nigella sativa* L.) seeds extract; All Hematoxylin and Eosin stain; Scale bars = 80 μm
